# A Cobotic, Digitally Controlled Schlenk‐line Unlocks Access to Elusive Lewis‐Base Stabilised Copper Bis(Disilylamides)

**DOI:** 10.1002/anie.202505408

**Published:** 2025-06-17

**Authors:** Nicola L. Bell, Marina Gladkikh, Cameron Fraser, Mostafa Elsayed, Emma Richards, Richard Drummond Turnbull

**Affiliations:** ^1^ School of Chemistry University of Glasgow Glasgow G12 8QQ UK; ^2^ School of Chemistry Cardiff University, Translational Research Hub Maindy Road Cardiff CD24 4HQ UK

**Keywords:** Automation, Copper(II), Digital chemistry, Inert atmosphere, Silylamide

## Abstract

Silylamides are important ligands in coordination chemistry for their ability to stabilise low coordination numbers and provide soluble, and even volatile, metal complexes. Such compounds provide valuable insights into the fundamental bonding and reactivity of their respective metals. Despite the wealth of homo‐ and heteroleptic hexamethyldisilazide complexes of divalent 3*d* ions (Sc‐Ni, Zn), attempts to access the corresponding divalent copper complexes have yielded only Cu(I) species. Herein, we demonstrate the stabilisation and isolation of a formally Cu(II) bis‐hexamethyldisilazide which was achieved by implementing novel digital chemistry tools. In order to successfully isolate (DMAP)Cu^II^(N{SiMe_3_}_2_)_2_ (DMAP = N,N‐dimethylaminopyridine), we investigated the roles of the co‐ligand and silylamide transfer reagent in the kinetics of its formation. Crucial to these studies was our newly developed “cobotic” Schlenk line which provides digital control of the atmosphere under which we conduct our highly reactive syntheses. In digitising Schlenk‐line handling, we have improved synthetic productivity by creating protocols for automated inertisation, solvent evaporation, liquid handling and crystallisation all while capturing reaction log data. Importantly, our Cu silylamide synthesis provides a case study showing that our cobotics approach allows for the discovery and isolation of unstable species which may remain elusive by traditional manual or fully autonomous methodologies.

## Graphical abstract

A Cobotic digital chemistry approach succeeds where manual synthesis failed. Herein, we present our new “AutoSchlenk” line which, coupled with other digital chemistry tools, was key to the synthesis and isolation of an unstable, and previously inaccessible, three‐coordinate Cu(II) hexamethylsilylamide complex.

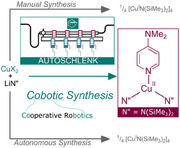

Recent years have seen dramatic advances in the automation of chemical syntheses, which may be described as a “digital chemistry revolution”.^[^ ^1^, ^2^, ^3^ ^]^ Much attention on this subject has focused on the development of closed‐loop “universal synthesisers” or “self‐driving labs”, which are able to conduct small‐scale reactions, analyse data and even make decisions about which further reactions to conduct.^[^ ^4^, ^5^, ^6^, ^7^ ^]^ Depending on one's perspective, this either liberates or relegates the human scientists to attend to other areas of work.^[^ ^8^ ^]^

An alternative strategy to capitalise on the value of automation involves cooperative robotics (cobotics).^[^ ^9^ ^]^ In this approach, robots and humans work seamlessly together, with automation of certain tasks and human input on others. This provides flexibility^[^ ^10^ ^]^ to change processes based on new information and allows each participant (robot and human) to play to their strengths.^[^ ^11^ ^]^ Cobotics provides a lower cost and potentially safer route to achieve a digital lab revolution, and arguably one that is already well underway with the use of peptide synthesisers, automated column chromatography and automated distillation (*via* smart rotary evaporators).^[^ ^12^ ^]^ In the field of frontline research and development, where full details of reaction conditions may not be known *a priori*, cobotics can provide the flexibility to change processes mid‐experiment based on new information, such as human observations. In organometallic chemistry, a field which has not yet been significantly influenced by automation,^[^ ^13^ ^]^ inherent hazards mean that cooperative automation may present a “best of both worlds” approach.

Recently, Bell, with others, has developed a universal synthesiser which includes an automated inert gas manifold and demonstrated its ability to undertake the full synthesis of a range of highly sensitive, previously known, organometallic complexes from across the periodic table.^[^ ^8^ ^]^ Whilst this proves that automation of sensitive chemistry is possible, this system, nonetheless, requires careful pre‐programming and prior knowledge of the whole synthetic process and, as such, is not ideally suited to the discovery and isolation of novel species. Universal synthesisers also require a significant investment of time, funds and training to set up which may hinder the exploitation of the advantages of lab digitalisation by some parts of the research community.

Herein, we describe a new low‐cost, cobotic lab tool, Autoschlenk, which provides the flexibility to discover and develop new synthetic routes ad hoc by streamlining the workflow for working under rigorously inert conditions whilst still prioritising continuous decision‐making freedom of the user. This flexible system is exemplified by the discovery and investigation of the long elusive Cu(II) bis(hexamethyldisilazide).

Homoleptic silylamide complexes are ubiquitous in coordination chemistry as highly soluble, halide‐free synthetic precursors which can be easy to access and purify at scale.^[^ ^14^, ^15^, ^16^ ^]^ For the divalent first‐row transition metals, bis(hexamethyldisilyl)amide complexes are known with Sc–Ni and Zn ions (Figure 1, top),^[^ ^17^, ^18^, ^19^, ^20^, ^21^, ^22^ ^]^ and these have been shown to be competent base metal catalysts for aliphatic C─H amination,^[^ ^23^ ^]^ hydroboration,^[^ ^24^ ^]^ depolymerisation,^[^ ^25^ ^]^ allylation^[^ ^26^ ^]^ and hydrosilylation.^[^ ^27^ ^]^ In 2015, Power et al. stabilised the long‐targeted NiN″_2_ [N″ = N(SiMe_3_)_2_] by coordination with solvent^[^ ^28^ ^]^ (L) which yielded products of the type (L)_n_NiN″_2_ (L = THF, n = 1; L = pyridine, n = 2) (Figure 1), extending the series of known early first‐row TM examples.^[^ ^29^, ^30^, ^31^, ^32^ ^]^ Divalent copper's growing use as an earth‐abundant catalyst^[^ ^33^, ^34^, ^35^ ^]^ and the ambiguity surrounding the mechanisms in such transformations^[^ ^36^, ^37^ ^]^ make the analogous amide complexes an attractive synthetic target. However, despite the successes with early 3d metals, the corresponding divalent copper complexes have eluded isolation to date, likely due to their significantly higher reduction potential [+0.34 V vs. < −0.26 V (Ni^II^)]. Upon treatment with bis(trimethylsilyl)amide reagents, Cu^II^X_2_ salts are known to reduce readily to yield the stable Cu^I^ tetramer [CuN″]_4_ (Figure 1).^[^ ^38^ ^]^ In 2016, Power et al. demonstrated the isolation of two‐coordinate Cu^II^{N(SiMe_3_)Dipp}_2_ (Dipp = C_6_H_5_‐2,6‐Pr*^i^*_2_ ) from disproportionation of Cu^I^X. Combined electron paramagnetic resonance (EPR) and density functional theory (DFT) analysis demonstrated its categorisation as a Cu(II) centre which was shown to be stabilised by dispersion effects, in contrast to other low‐coordination number copper compounds which are better described as Cu(I) with a ligand based radical.^[^ ^39^, ^40^, ^41^ ^]^

**Figure 1:**
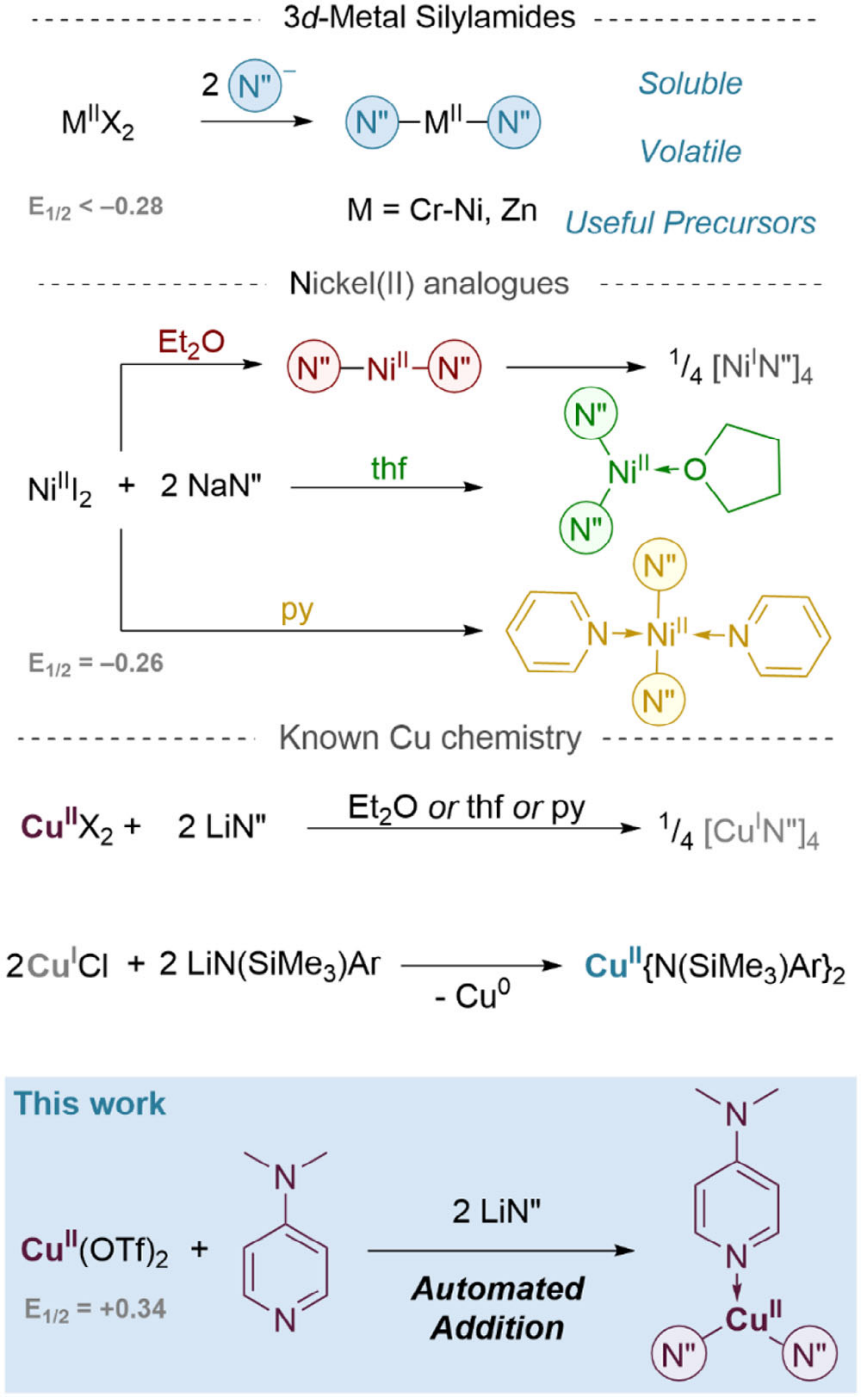
A summary of the known first‐row transition metal silylamide complexes and the relative reduction potentials of their respective M(II) ions. Notably nickel analogues can be stabilised by solvent coordination. The stability of the first‐row transition metal bis(hexamethyldisilazide) complexes closely follows the reduction potential of the divalent oxidation state (grey). Ar = 2,6‐diisopropyl, X = Br; N″ = N(SiMe_3_)_2_.

Herein, we report the discovery of a stable Cu(II) bis(hexamethyldisilylamide) by a human scientist and its ultimate synthesis and isolation made possible by application of cobotic lab tools, including the Autoschlenk.

A Schlenk line, or gas/vacuum manifold, consists of two glass tubes or manifold lines, one of which is kept under reduced pressure by application of a vacuum pump while the other is maintained under positive pressure by the introduction of a gas (usually N_2_ or argon). A set of between four to six pairs of taps allows the connection of flasks to the manifold *via* flexible tubing.^[^ ^42^ ^]^ Autoschlenk (Figure 2, top) digitises actuation of these taps to streamline their operation, allowing for automation, data recording and fine control. Application of vacuum or gas to a reactor vessel is based upon the position of linear actuators which move to open and close taps on the manifold. 3D‐printed tap barrels appended with chemically resistant FKM o‐rings ensure a gas‐tight seal at each tap. Actuation is controlled using the Arduino framework, deployed *via* PlatformIO, on a bespoke printed circuit board (PCB) housing an ATMega processor which can actuate up to 15 motors at once. Fine control of each actuator is possible, thanks to potentiometer feedback allowing the system to be calibrated to log the position of all actuators (“Taps”) at any given time. This allows for the partial opening of taps as required by functions such as “*Evaporate*” and “*Transfer*” (*vide infra*).

**Figure 2:**
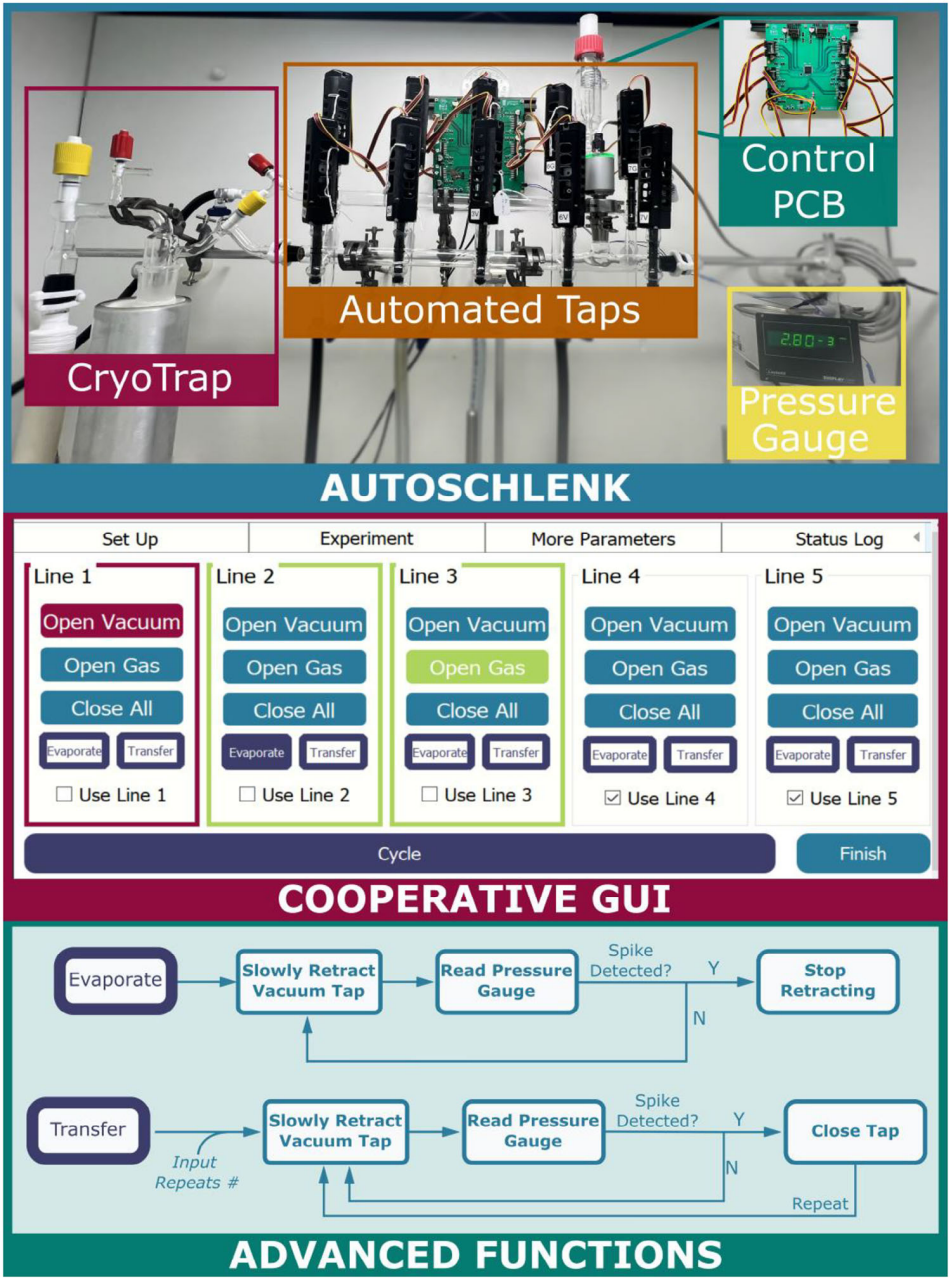
Top: Autoschlenk; middle: Raspberry Pi GUI; bottom: Workflow for newly developed functionalities which exploit digital vacuum gauge feedback.

Digital control of the device can be conducted *via* three routes of increasing flexibility and decreasing interactivity: 1) Raspberry Pi touchscreen control *via* Pi‐GUI (graphical user interface); 2) PC GUI; 3) Direct programmatic control (*via* either Python or C++) for integration with other automation hardware. The Raspberry Pi interface (Figure 2, middle), developed using PyQT, allows for touchscreen operation of the Autoschlenk as a standalone unit in the lab, dramatically increasing the accessibility of this type of chemical manipulation. The Pi interface provides the option to “*Open*” and “*Close”* each of the taps individually as well as selecting several taps for advanced protocols such as “*Cycle”*, *“Evaporate”*, “Transfer” and “*Concentrate”* (Figure 2, bottom), the details of which will be discussed below. A related GUI which can be operated *via* a windows laptop or equivalent provides additional control options including monitoring of logging data and varying the speed of tap opening/closing, controlling tap opening percentage. Finally, as the underlying GUI code is Python based, it is possible to integrate this system with other digital control systems such as Labview.

Alongside simple unit operations of application of vacuum or gas to each reactor line, the key processes undertaken manually on a Schlenk line include cycling or inertisation, which removes air from the reactor in question; evaporation, which allows for solvent distillation into a cryogenic trap; transfer, movement of liquids from one flask to another (e.g., cannulation) and concentration, a crystallisation protocol.

In developing this system, we have included four important protocols to digitise each of these key unit operations. The *Cycle* function facilitates automated inertisation of one, several or all of the reactor lines connected to the system. This is undertaken by the sequential opening of vacuum taps followed by opening the gas taps, both for a user‐defined period, before repetition of this process in triplicate or more. The *Evaporate* step allows for opening of a vacuum tap to a reactor vessel containing a volatile liquid for distillation into a cryogenic trap (Figure 2, bottom). Importantly, this method incorporates pressure feedback *via* a Leybold TTRN9 gauge to prevent solvent bumping. In this protocol the vacuum tap is opened slowly until a pressure spike is detected in the reduced pressure line at which point the tap progression is stopped to allow for slow evacuation of the reactor which minimises solvent bumping. The *Transfer* step enables cannulation of liquid species between two reactors (e.g., A & B) without the requirement for a liquid‐handling pump. The opening of a tap to the positive pressure/gas line to reactor A is then followed by a brief opening of reactor B to the reduced pressure line (controlled by detection of a pressure spike as described for *Evaporate*). The result is a pressure differential sufficient to move liquids between the two flasks. This is particularly useful in filtration procedures whereby transfer can take extended periods of time. Finally, the *Concentrate* protocol allows for sequenced opening and closing of the vacuum tap of an associated reactor. For highly soluble materials, it is often desirable to achieve dissolution in the minimum solvent possible for crystallisation. Simple evaporation steps can result in solid material drying out, and even degrading, on the sides of the flask as a residue is left behind when the solvent meniscus recedes. The “*Concentrate”* protocol provides the opportunity for the reactor to equilibrate at reduced pressure, which results in internal condensation of solvent vapour on flask walls, effectively washing the solid material back into solution (See Figure S28 for more detail). Below, we detail how each of these methodologies were used in the synthesis of our copper bis(silylamide).

Inside an inert‐atmosphere glovebox, two flasks were charged with Cu^II^(OTf)_2_ and DMAP (2 equiv.), respectively. These two flasks as well as a toluene solvent ampoule were connected to the Autoschlenk manifold and the Raspberry Pi touchscreen was used to initialise the *Cycle* protocol on three reactor lines (Figure 3). After automated cycling completed, the flasks were manually opened under positive gas pressure and Subaseal septa were appended to each with stainless steel cannulae connecting the flasks. First, solvent was transferred into each flask by opening and closing the vacuum and gas taps *via* the Pi touchscreen to allow for close degree of control over the volume transferred (*ca* 10 mL). Next, the *Transfer* protocol was implemented by applying positive pressure to the flask containing a toluene solution of DMAP and a slight negative pressure to the Cu(OTf)_2_ flask. The resultant solution was sealed and sonicated for *ca* 4 h, whereupon the suspension turned from purple to lime green. The *transfer* protocol was used again to facilitate cannula filtration of the insoluble Cu(DMAP)_2_(OTf)_2_ product (**1a**). Cu(DMAP)_4_(OTf)_2_ (**1b**) was prepared similarly by adapting literature procedures for the Autoschlenk.^[^ ^43^ ^]^

**Figure 3:**
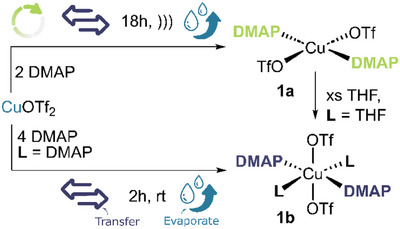
Synthesis of DMAP adducts of copper triflate salts (**1a‐b**) using Autoschlenk functions Cycle, Transfer and Evaporate.

Manual treatment of a THF solution of Cu^II^(OTf)_2_ with KN″ (2 equiv.) yields an immediate red colour at the point of addition which fades in <1 s. This instability would likely practically preclude discovery of the transient red “lead” species by most autonomous systems. Alternatively, treatment of a suspension of **1b**, containing the strong Lewis base DMAP, with a toluene solution of KN″ (2 eq.) results in a red solution which fades within minutes. NMR spectroscopy, run within minutes of mixing, characterised the ultimate product of both reactions as colourless [Cu^I^N″]_4_ as observed in a similar reaction by Maverick *et al*.^[^ ^38^ ^]^

Despite this, we hypothesised that the short‐lived red species could represent the elusive [(L)Cu^II^N″_2_] (L = THF, DMAP) akin to the solvated examples of other first‐row transition metals.^[^ ^32^ ^]^ Broad resonances in the ^1^H NMR spectrum of mixtures of Cu(DMAP)_n_(OTf)_2_ and MN″ at 5.44 (36H) and −1.45 (6H) ppm indicated the presence of a paramagnetic Cu(II) species, which was further characterised by EPR, UV‐Vis and DFT calculations (see SI).

Both formation and degradation of the red species were found to be faster when mixing THF solutions of the reagents, while stirring suspensions of the reagents in hexanes allowed for the persistence of the red colour for days after an induction period of several minutes. Toluene was also found to be a suitable solvent for this reaction, with minimal degradation taking place over the first few hours (Table 1). Varying the alkali metal cation for the silylamide reagent from potassium to lithium was shown to yield significantly less degradation to Cu(I) and addition of excess DMAP was also found to slow the rate of reduction, providing increased conversion to **2** (Figure 4).

**Table 1:** Survey of the effect of key reaction parameters on the conversion to **2** as well as the ratio of **2**:[Cu^I^N″].

C u(DMAP)_n_ (OTf)_2_ n =	[MN″] (1.8 eq.)	Solvent [25mM(Cu)]	[2] (%)	[2]:[Cu^I^]
2	KN″	PhMe	32%	0.65
2	NaN″	PhMe	37%	1.3
2	LiN″	PhMe	45%	2
4	LiN″	PhMe	49%	4.5
2	LiN″	Hexane	37%	1.6
3	LiN″	Hexane	38%	2.3
4	LiN″	Hexane	18%	3
4	LiN″	PhMe^a)^	55%	7
4	LiN″ ^b)^	PhMe	57%	17
4	LiN″ ^c)^	PhMe	74%	23
4	LiN″ ^d)^	PhMe	94% (88%)	60

^a)^Temperature −35 °C; ^b)^ Addition rate 0.18 equiv. min^−1^ (manual dropwise); ^c)^ Addition rate 0.022 equiv. min^−1^ (dropping funnel); ^d)^ Addition rate 0.017 equiv. min^−1^ (automated syringe pump).

In order to gain more insight, kinetic UV‐vis studies were undertaken with a large excess of DMAP providing both a stabilising effect on **2** and a steady state for our experiments. UV‐Vis spectra, gathered immediately upon mixing of THF solutions (<10 s after mixing) of the reagents, show a peak at 508 nm corresponding to the raspberry‐red solution observed. Kinetic measurements determined degradation to be second order (Figure S4, Equation 1) leading us to hypothesise that the formation of the “ate”‐salt (S)M[Cu(II)N″_3_] (Figure 4a, S = THF, DMAP, M = Li, Na, K), which is known for the nickel(II) analogue,^[^ ^27^, ^44^, ^45^ ^]^ and other transition metals.^[^ ^46^ ^]^ We propose that the formation of (S)M[Cu(II)N″_3_] is promoted by the presence of unreacted MN″ in solution and results in immediate degradation to [Cu^I^N″]_4_, possibly due to Jahn‐Teller‐induced destabilisation of the *d* ^9^ trigonal planar Cu(II) centre.^[^ ^47^, ^48^, ^49^ ^]^

**Figure 4:**
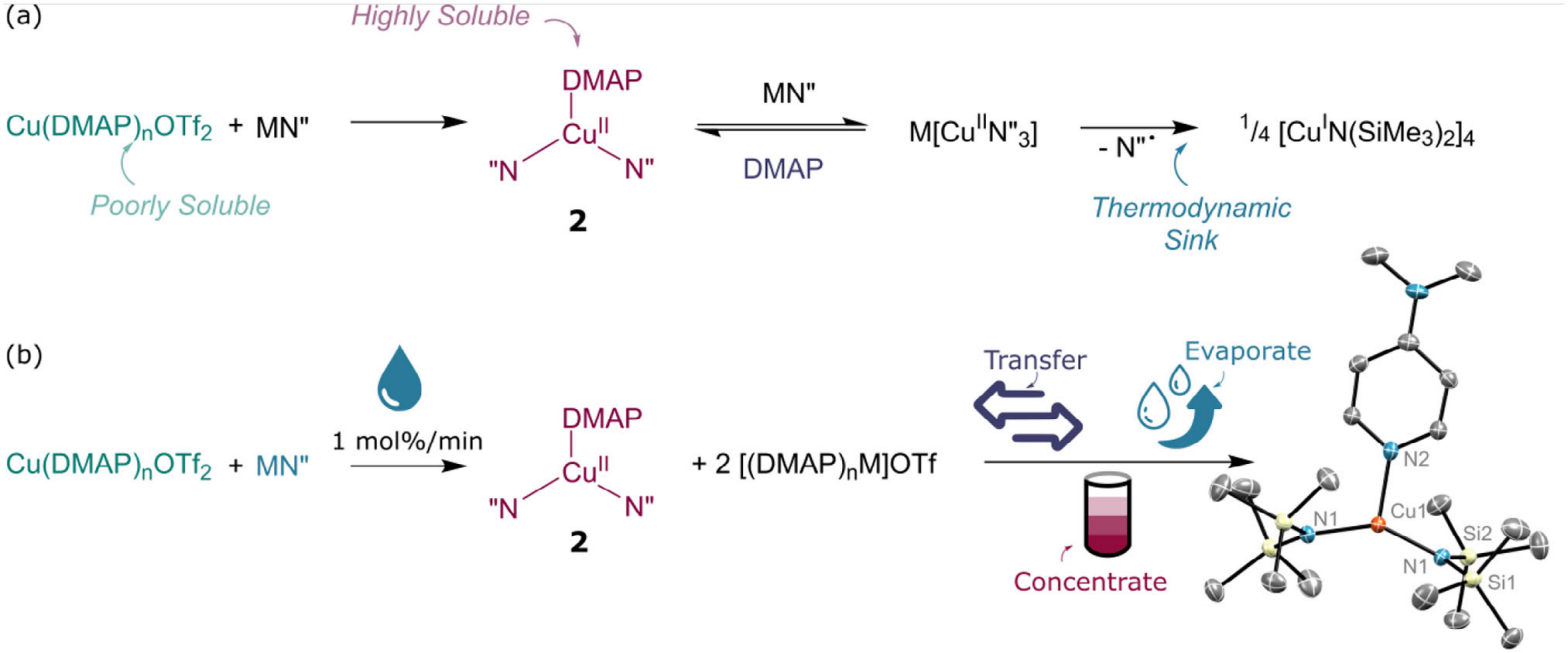
a) Proposed mechanism for the synthesis and degradation of DMAPCuN”_2_ (**2**). b) Cobotic method of synthesis and isolation of **2** showing the X‐ray crystal structure of isolated material. H‐atoms removed for clarity. Cu1‐N2: 1.8819(18) Å; Cu1‐N1: 1.952(3) Å



(1)

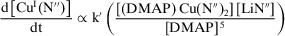




Together, these data presented a synthetic challenge: one of the reactants in this transformation catalyses the degradation of the desired product. Importantly, removal of solvent from any mixture of LCuN″_2_ and MN″ (i.e., before full consumption of MN″) accelerated degradation as the relative concentration of MN″ increases.

The key synthetic variable for stabilisation of (L)Cu^II^N″_2_ (L = DMAP) was therefore proposed to be the rate of addition of LiN″, which must be kept as slow as possible to limit the formation of Li[CuN″_3_] (Figure 4b). Thus, isolation of (DMAP)Cu^II^N″_2_ was ultimately achieved by cobotic automation of the synthetic process using our Autoschlenk coupled with a liquid handling pump. Utilising the Autoschlenk manifold, two reactors containing a suspension of Cu(DMAP)_2_(OTf)_2_ (**1a**) and a solution of LiN″ (2 equiv.), respectively, were inertised automatically using the *Cycle* function. Ultraslow addition of a toluene solution of LiN″ (208 µL min^−1^, *ca* 20 mL) to a suspension of Cu(DMAP)_2_(OTf)_2_ followed by stirring for 3 h at room temperature resulted in the formation of a deep raspberry‐red solution containing a dark precipitate. After this, the Autoschlenk *Transfer* protocol was used to affect filtration. Next, the *Evaporate* protocol engaged to remove the toluene solvent, smoothly, yielding a deep red solid. Dissolution of the residue in the minimum volume of pentane followed by cooling to −90 °C did not yield crystalline material; however, application of the *Concentrate* protocol on the AutoSchlenk line to this solution ultimately provided X‐ray quality crystals of (DMAP)Cu^II^N″_2_ in high yield (88%). Notably, two key features of our cobotic setup allowed for this isolation which was not possible manually in our hands: 1) Ultraslow addition *via* liquid handling pumps under a rigorously inert atmosphere and 2) Crystallisation using the *Concentrate* protocol.

In summary, we have isolated a copper(II) bis(hexamethyldisilazide) complex for the first time by stabilisation with a N,N‐dimethylaminopyridine co‐ligand. Through UV‐vis kinetic studies, we demonstrate that the degradation of this species occurs rapidly in the presence of MN″ due to the formation of a proposed short lived “ate”‐salt which reductively degrades to Cu^I^. Synthesis of (DMAP)Cu^II^N″_2_ was achieved only by digitally controlled ultraslow addition of the silylamide reagent to the reaction mixture alongside application of the advanced cobotic functions of our new digital Schlenk line (Autoschlenk).

Autoschlenk provides advanced functionalities (*Cycle*, *Evaporate*, *Transfer*, *Concentrate*) *via* manifold taps which incorporate pressure gauge feedback in their control and allows for various modes of interactivity enabling non‐expert users to incorporate automation into their workflows. For the isolation of **2**, the “*Concentrate*” function, in particular, provided a novel strategy to facilitate the ultimate crystallisation of this highly pentane‐soluble species.

The results herein demonstrate the “best of both worlds” approach of cooperative robotics. Our first discovery of this short‐lived intermediate by observation of the red flash in solution upon mixing of THF solutions of Cu(OTf)_2_ and KN may not have been observable on the timescale of many autonomous analytical routines; however, the product would not have been isolable without the cobotic automation tools detailed herein. Together, this work makes a case for the adoption of low‐cost cobotic strategies in chemical synthesis and research more widely in order to lower the barrier to entry and thus democratise lab digitalisation whilst improving laboratory accessibility and productivity.

## Supporting Information

The supplementary information document attached includes full synthetic and analytical details, derivation of Equation 1, NMR spectra, EPR spectra and DFT computational results. X‐ray crystal structures for CSD 2409514, 2409515, 9409516 are also depicted in the SI file while the full data sets are available from ccdc.cam.ac.uk/structures. Build instructions and a bill of materials for the Autoschlenk as well as an explanation of the code stack structure are also in the SI document. Full schematics for the PCB board, Firmware and GUI software can be found at github.com/Bell‐Group‐Glasgow and is provided subject to a Creative Commons CC‐BY‐NC license.

## Conflict of Interests

The authors declare no conflict of interest.

## Supporting information



Supporting Information

Supporting Information

## Data Availability

The data that support the findings of this study are available in the Supporting Information of this article.
