# Supplementary material for: A Cobotic, Digitally Controlled Schlenk‐line Unlocks Access to Elusive Lewis‐Base Stabilised Copper Bis(Disilylamides)
Source: Angew Chem Int Ed Engl. 2025 Jun 17;64(33):e202505408. doi: 10.1002/anie.202505408 (PMC12338420; doi:10.1002/anie.202505408)
Supplement: Supplementary file 1 — Supporting Information [file ANIE-64-e202505408-s001.docx]

A Cobotic, Digitally-Controlled Schlenk-line Unlocks Access
to Elusive Lewis-base stabilised Copper(II) Bis(Disilylamides)

Nicola L. Bell^[a]^*, Marina Gladkikh^[a]^, Cameron Fraser^[a]^, Mostafa Elsayed^[a]^, Emma Richards and Richard Drummond Turnbull^[a]^

[a] School of Chemistry
University of Glasgow
Glasgow, G12 8QQ, UK
E-mail: [Nicola.Bell@glasgow.ac.uk](mailto:Nicola.Bell@glasgow.ac.uk)

[b] School of Chemistry

Cardiff University

Main Building

Park Place

Cardiff

CD10 3AT

Table of Contents

[1 Synthesis 2](#_Toc184896092)

[1.1 General Experimental 2](#_Toc184896093)

[1.2 Preparation of Starting Materials 3](#_Toc184896094)

[1.2.1 [(DMAP)_2_Cu(OTf)_2_] 3](#_Toc184896095)

[1.2.2 [(DMAP)_4_CuOTf_2_] 3](#_Toc184896096)

[1.3 Synthesis, Analysis and Stability Tests of Copper(II) Silylamide 4](#_Toc184896097)

[1.3.1 Synthesis of [(DMAP)Cu{N(SiMe_3_)_2_}_2_] 4](#_Toc184896098)

[1.3.2 UV/Vis Experimental Protocol 5](#_Toc184896099)

[1.3.3 UV/Vis Experimental Data 6](#_Toc184896100)

[1.3.4 UV/Vis Data - Kinetic Experiments 7](#_Toc184896101)

[1.3.5 Optimization Table (Table 1) Protocol 12](#_Toc184896102)

[1.4 NMR Spectra 13](#_Toc184896103)

[1.4.1 H^1^ NMR Spectrum of [(DMAP)_2_Cu(OTf)_2_] at 25 °C. 13](#_Toc184896104)

[1.4.2 H^1^ NMR Spectrum of [(DMAP)_2_Cu(OTf)_2_] at 50 °C 13](#_Toc184896105)

[1.4.3 ^1^H NMR Spectrum of [(DMAP)_4_CuOTf_2_] 14](#_Toc184896106)

[1.4.4 ^1^H NMR Spectrum of [(DMAP)Cu{N(SiMe_3_)_2_}_2_] 14](#_Toc184896107)

[1.5 X-ray Diffraction Data 15](#_Toc184896108)

[2 Autoschlenk 27](#_Toc184896109)

[2.1 PCB Control Board 27](#_Toc184896110)

[2.2 Bill of Materials 27](#_Toc184896111)

[2.3 Build Guide for Actuators 28](#_Toc184896112)

[2.4 Code 31](#_Toc184896113)

[2.5 TOC Artwork 34](#_Toc184896114)

# Synthesis

## General Experimental

All manipulations were performed under an argon atmosphere using appropriate Schlenk line and glovebox techniques. The latter were conducted in an MBraun LabStar glovebox operating at internal H_2_O and O_2_ concentrations of <0.5 ppm. Unless otherwise specified, all transformations referring to the use of a Schlenk line are carried out on the Autoschlenk system appended with a Leybold TRIVAC D4B vacuum pump and a Yorlab Overpressure Bubbler operating at 1.3 bar overpressure. Autoschlenk was operated through a bespoke GUI running on a DELL 41 laptop or a Raspberry Pi with a 7" LCD touch screen. The manifold was calibrated prior to the start of each experiment using the ‘Calibrate’ function. Where mentioned below standard Schlenk line techniques such as line and glassware inertisation via cycling, cannulation/cannula filtration and solvent evaporation were carried out using the ‘Cycle’, ‘Transfer’ and ‘Evaporate’ protocols respectively. ‘Open Gas’ and ‘Open Vacuum’ commands were used as necessary.

All reagents and solvents were obtained from commercial suppliers and used without purification unless otherwise specified. Anhydrous THF, toluene and Et2O were obtained from a PureSolv 500 MD Solvent Purification System by Innovative Technology Inc., handled under inert atmosphere, degassed and stored over activated 3 Å molecular sieves. Hexanes, Pentane and n-hexane solvents were freeze-pump-thaw degassed in triplicate and stored over activated 3 Å molecular sieves for at least 24 h prior to use. Anhydrous d8-THF and d6-benzene were refluxed over potassium metal for 16 h, freeze-pump-thaw degassed in triplicate, and vacuum distilled prior to storage under dry argon in a glovebox. Glassware was dried overnight at 150 °C and allowed to cool under a dynamic vacuum (ca. 10−3 mbar) prior to commencing work.

Experiments requiring a liquid handling pump (LHP) were carried out using an Advanced Microfluidics LSPone Syringe Pump with a 1 mL syringe installed and operated via open-source LSPone generated Python scripts. All reactions using the LHP were setup using oven-dried PTFE tubing (Diba, 008T32-150-100, 3.2 mm OD x 1.5 mm ID) appended with Idex PFA flangeless fittings (XP-345) while unused ports on the pump were sealed off using ETFE blanking plugs (Idex, P-311). All systems using the LHP were purged with 20 mL of the reaction solvent during experiment setup.

^1^H and ^13^C NMR spectra were recorded on 400 MHz Ultrashield or Ascend Bruker spectrometers with BBO probe Avance III HD consoles. Chemical shift values were recorded in parts per million (ppm) and referenced to the specified deuterated solvent—either C_6_D_6_ or d_8_-THF. NMR spectra were processed using MestReNova 14.3.0 software. Shifts for paramagnetic species are reported only where resonances were identifiable.

Sample for EPR measurement was loaded into a Young’s EPR tube under an N2 atmosphere in a glovebox (~ 10 mg, 100:50 uL toluene:fluorobenzene). The X-band CW EPR measurements (T = 120 and 298 K) were performed on a Bruker EMX spectrometer utilizing an ER 072 magnet/ ER 081 power supply combination (maximum field 0.6 T), an ER4119HS resonator, operating at 100 kHz field modulation, 0.3 mT modulation amplitude (which avoided over-modulation) and 10 mW microwave power (under the saturation limit).

Simulations of all EPR spectra were performed using the garlic or pepper functions within the Easyspin toolbox for Matlab.[1]

All UV/Vis measurements were carried out on a HORIBA Duetta Bio Fluorescence and Absorbance spectrometer using the “Absorbance” function available through the EZSpec software. Experiments were performed in quartz cuvettes with a 10 mm path length, equipped with a 10 mm fine-threaded J. Young PTFE stopcock. Obtained experimental data was processed using internally generated Python scripts available on [GitHub](https://github.com/Bell-Group-Glasgow/autoschlenk).

Elemental analyses were conducted by O. McCullough at Elemental Analysis Services Team, Science Centre, London Metropolitan University. Air-sensitive EPR studies were carried out by Dr. E. Richards at the University of Cardiff. MS measurements were carried out by G. Rossi at the University of Glasgow.

Single crystal X-ray diffraction data was collected at 150(2) K on a Rigaku XtaLAB Synergy R diffractometer equipped with a graphite monochromator (λMo-Kα = 0.71073 Å) of a micro-focus sealed X-ray source (50 kV, 24.0 mA). Data collection and reduction were performed using the CrysAlisPro software package and structure solution and refinement were carried out with SHELXT 2018/2 and SHELXL-2019/3 via Olex2 v1.5. All the non-hydrogen atoms (including those disordered) were anisotropically refined.

## Preparation of Starting Materials

### [(DMAP)_2_Cu(OTf)_2_]

Scheme S1: Synthesis of bis(dimethylaminopyridine)cupric triflate (1a)

To an ampoule equipped with a 10 mm J. Young PTFE stopcock was added 4-dimethylaminopyridine (339.1 mg, 2.776 mmol), copper(II) triflate (508.0 mg, 1.405 mmol), and toluene (~25 mL). The flask was then sealed and sonicated for 18 h at 40 °C, during which time the solution turned from lavender purple to pastel green. Following sonication, the green solid was isolated by cannula filtration, the resultant solids were washed with hexanes (~25 mL) and the final product was dried *in vacuo* to yield copper(II) triflate bis-4-dimethylaminopyridine, as a fine, pastel green powder (1568.1 mg, 2.588 mmol, 93 %). MS(ESI+) m/z 226.03 (M-2[OTf]); ^1^H NMR (THF-*d_8_*, 400 Hz, 25 °C) *d* 36.5 (1.88 H, br. s, w_1/2_ » 4,000 Hz), 0.50 (6H, br. s, w_1/2_ = 113.0 Hz), 0.22 (8.73 H, br. s, w_1/2_  = 70.5), -0.35 (1.49H, br. s, w_1/2_  = 59.7 Hz); ^1^H NMR (THF-*d_8_*, 400 Hz, 50 °C) *d* 33.72 (1H, br s, w_1/2_ » 3000 Hz), 0.53 (br. s, 12 H, w_1/2_  = 185.0 Hz ) ppm. UV/Vis (THF, 5 mM): 300 nm (347 M^-1^ cm^-1^). Anal. Calcd. for C_16_H_20_CuF_6_N_4_O_6_S_2_ requires: C, 32.82, H, 3.89, N, 9.01; found C, 32.87, H, 3.26, N, 9.08.

### [(DMAP)_4_CuOTf_2_]

Scheme S2: Synthesis of tetrakis(dimethylaminopyridine)cupric triflate (1b)

4-dimethylaminopyridine (968.0 mg, 7.923 mmol) and copper(II) triflate (720.0 mg, 1.991 mmol) were dissolved in toluene (35 mL) and stirred for 18 h at room temperature. The solution turned lavender purple and a fine precipitate of the same colour evolved. The solution was filtered, and the resultant solids were washed with toluene (2 x 20 mL). The final product was dried *in vacuo* to yield [Cu(DMAP)₄(OTf)₂] as a fine, lavender powder (1052.6 mg, 1.238 mmol, 63%). Analytical data on the isolated material was consistent with literature reported values.^[2]^

## Synthesis, Analysis and Stability Tests of Copper(II) Silylamide

### Synthesis of [(DMAP)Cu{N(SiMe_3_)_2_}_2_]

Scheme S3: Synthesis of (N,N-dimethylaminopyridine)bis(hexamethyldisilazido)copper(II) (2).

To a suspension of [(DMAP)_4_CuOTf_2_] (1.18 mmol, 1 equiv.) in toluene (0.4 M) was added a solution of LiN(SiMe_3_)_2_ (2.35 mmol, 2 equiv.) in toluene (0.8 M) over 120 minutes (2 mol%/min or 0.01 equiv./min) using a liquid handling pump (LHP) during which time the solution turned deep red. The resulting suspension was stirred for another 3 h before cannula filtration to remove LiOTf salts. Removal of the solvent *in vacuo* yielded a red residue which was extracted into pentane (*c.a.* 30 mL). Further filtration and concentration of the pentane extracts was undertaken until crystallization was observed (*ca.* 5 mL), whereupon the solution was cooled in an ethanol/liquid N_2_ bath to yield red crystals of [(DMAP)Cu(N”)_2_] (88%). MS(ESI+) m/z 505.0 (M+) ^1^H NMR (C_6_D_6_, 400.23 Hz) *δ* 54.5 (br. s, 2 H, w_1/2_ = 2616 Hz ), 5,44 (br. s, 36 H, w_1/2_  = 169.7 Hz), 2.19 (br. s, 2H, w_1/2_  = 158.9 Hz), -1.45 (br. s, 6H, w_1/2_  = 82.6 Hz) ppm. UV/Vis (THF, 1.5 mM): 508 nm (1,231 M^-1^ cm^-1^). Anal. Calcd. for C_19_H_45_CuN_4_Si_4._0.5(C_7_H_10_N_2_) requires: C, 47.61, H, 9.06, N, 12.34; found C, 47.33, H, 8.91, N, 12.13.

## UV/Vis Experimental Protocol

All UV/Vis data were gathered using general procedure outlined below. Reactant solutions of a known concentration (CuOTf_2_: 3.33 mM; LiN”: 33.5 mM or 23.9 mM) were prepared using 5 mL and 10 mL volumetric flasks inside the glovebox. To a quartz cuvette fitted with an integral 10 mm J. Young PTFE stopcock and reagent reservoir, the appropriate volume of each solution was added so that the total volume of reaction solution equaled 5.5 mL. Both the CuOTf_2_/1a/1b solutions and solution of DMAP were mixed in the cuvette segment of the glassware, while the MN(SiMe_3_)_2_ solutions were placed in the separated reagent reservoir. The solutions were then vigorously mixed immediately before starting data collection. A total of 160 scans were gathered for each data set (equivalent to a scan running every 40 s).

Table S1: Table of amounts used for UV-vis studies showing the number of millimoles (mmol) used, the concentration (mM) of the stock solution used and the volume (mL) of stock solution added for each run as well as the mass (mg) of excess DMAP added. Also shown is the rate as obtained from the slope of a straight line derived from a plot of 1/Absorbance vs time for each run.

| Run | Cu(DMAP)_2_OTf_2_ | | | LiN” | | | DMAP | | |  | Obs. Rate |
| --- | --- | --- | --- | --- | --- | --- | --- | --- | --- | --- | --- |
|  | mmoles | Conc. | Vol. | mmoles | Conc. | Vol. | mmoles | Mass | Vol. |  |  |
| 1 | 7.5 | 3.33 | 2.0 | 17.8 | 33.5 | 0.53 | 150 | 18.3 | 2.7 |  | 1.23 |
| 2 | 8.5 | 3.33 | 2.6 | 20.4 | 24.0 | 0.85 | 170 | 20.8 | 2.1 |  | 0.95 |
| 3 | 10 | 3.33 | 3.0 | 24 | 24.0 | 1.0 | 200 | 24.4 | 1.5 |  | 0.36 |

## UV/Vis Experimental Data

**
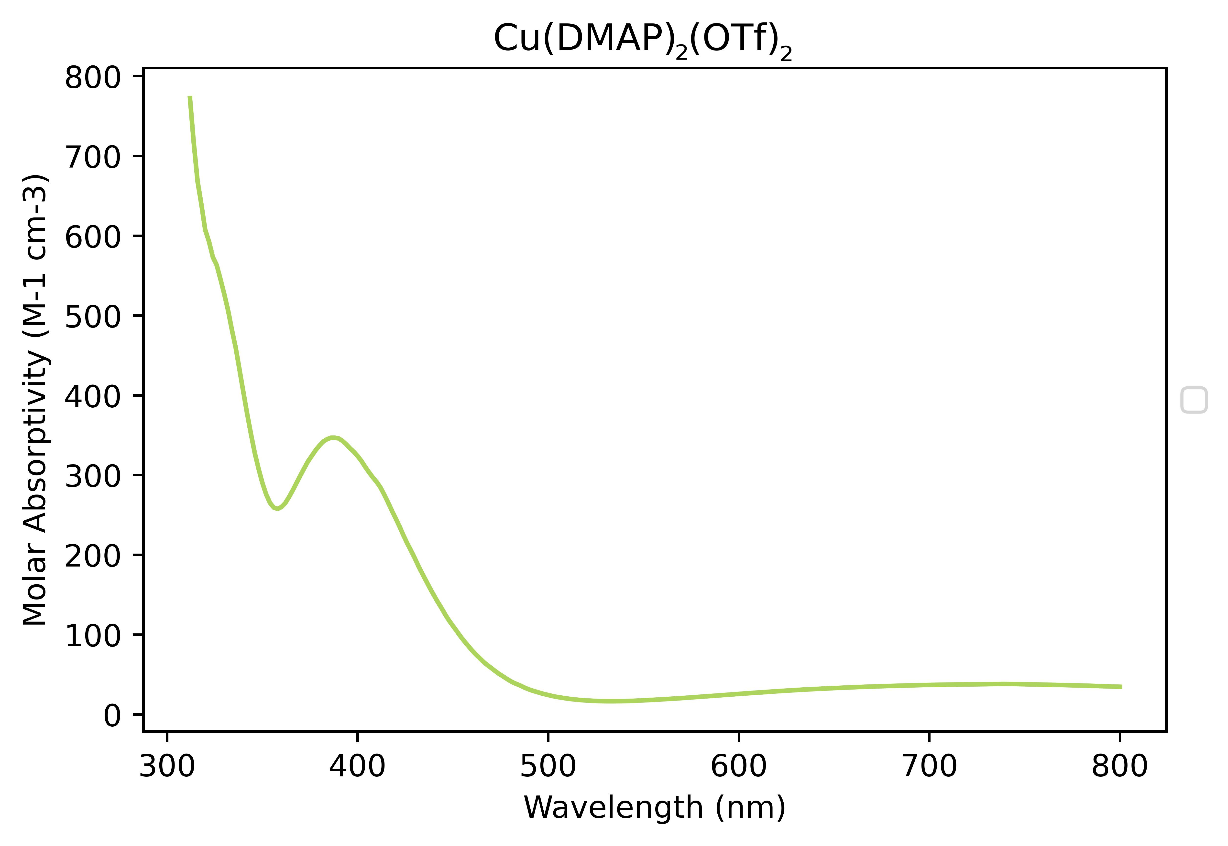
**

**Figure S1**: Molar absorption coefficient vs. wavelength graph of Cu(DMAP)_2_(OTf)_2_ (5.1 mM) in THF.

**
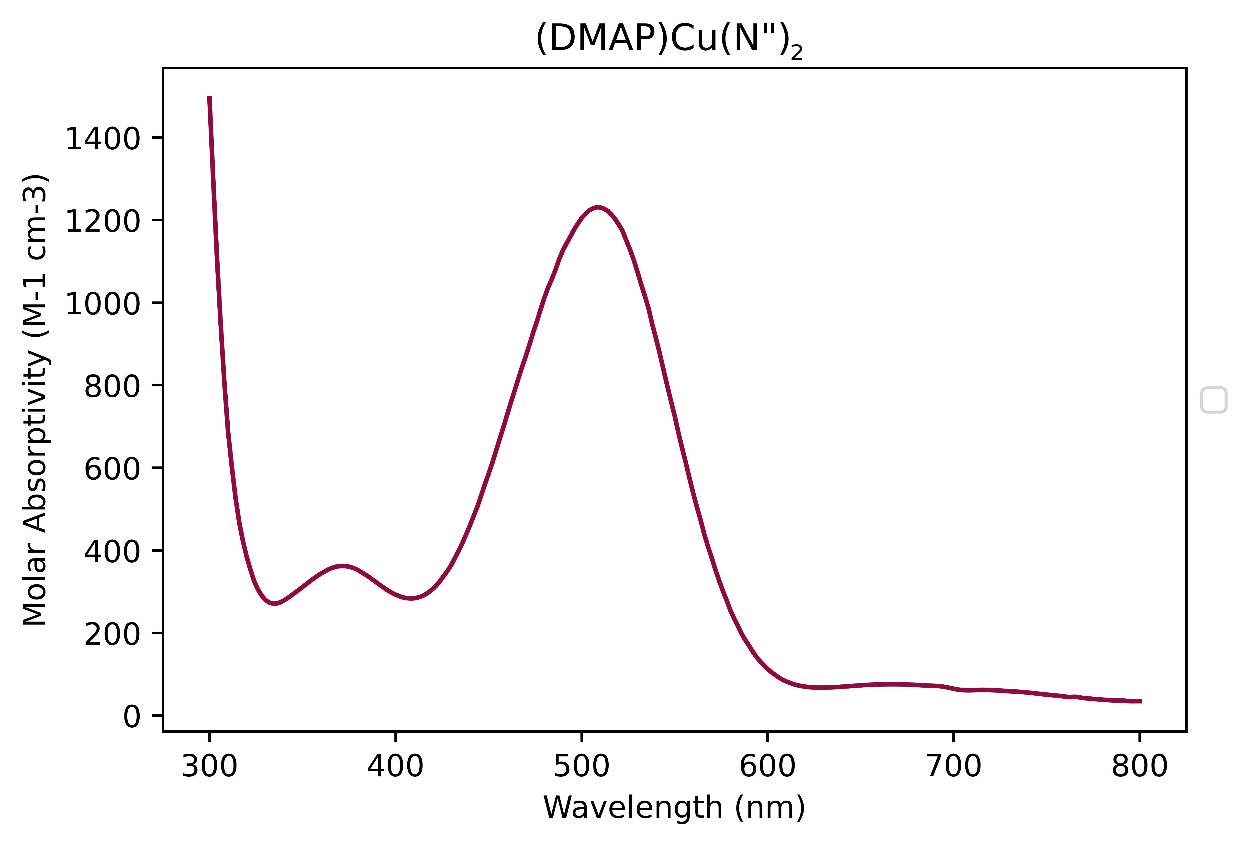
**

**Figure S2**: Molar absorption coefficient vs wavelength graph [(DMAP)Cu{N(SiMe_3_)_2_}_2_] (1.8 mM) in THF.

## UV/Vis Data - Kinetic Experiments


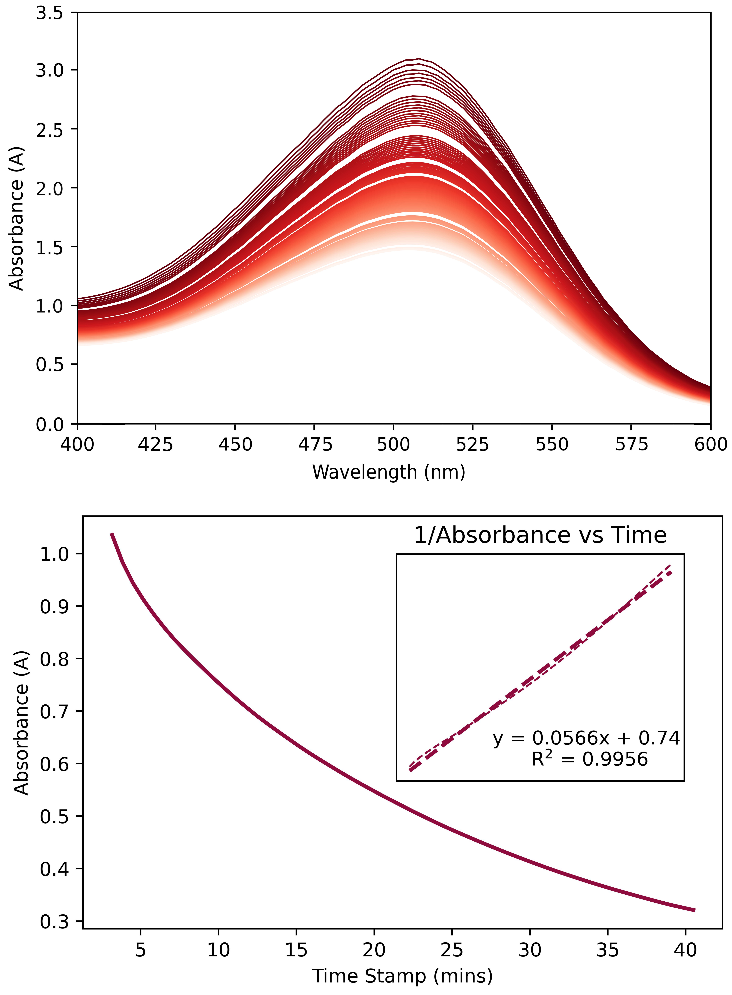


Figure S3: Plot of Absorbance vs wavelength of [(DMAP)Cu{N(SiMe_3_)_2_}_2_] (10 µM) in THF over time

**
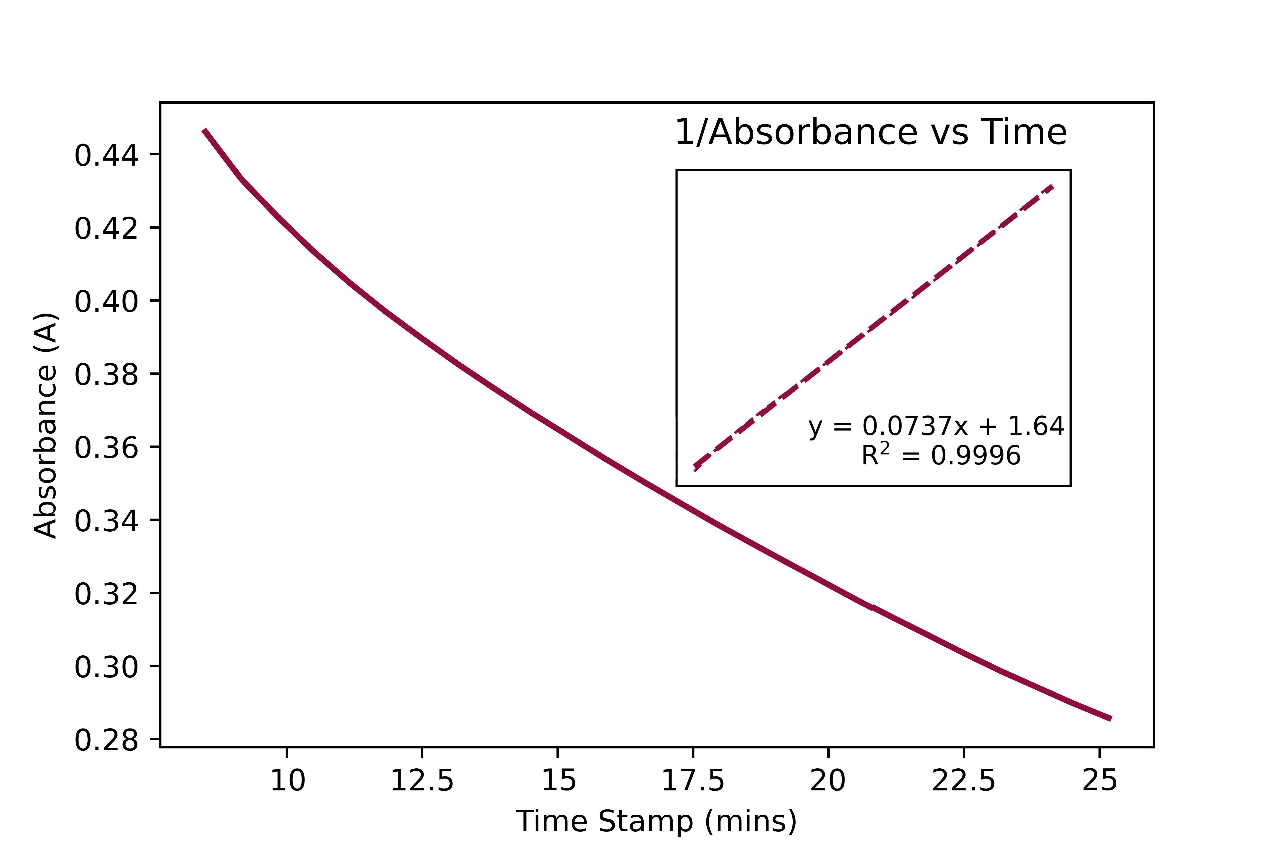
**

Figure S4: Plot showing how A_max_ changes when Cu(DMAP)_2_(OTf)_2_ at 7.5 µmol/L reacts with MN”. Its respective linear trendline when 1/A_max_ is also shown.

**
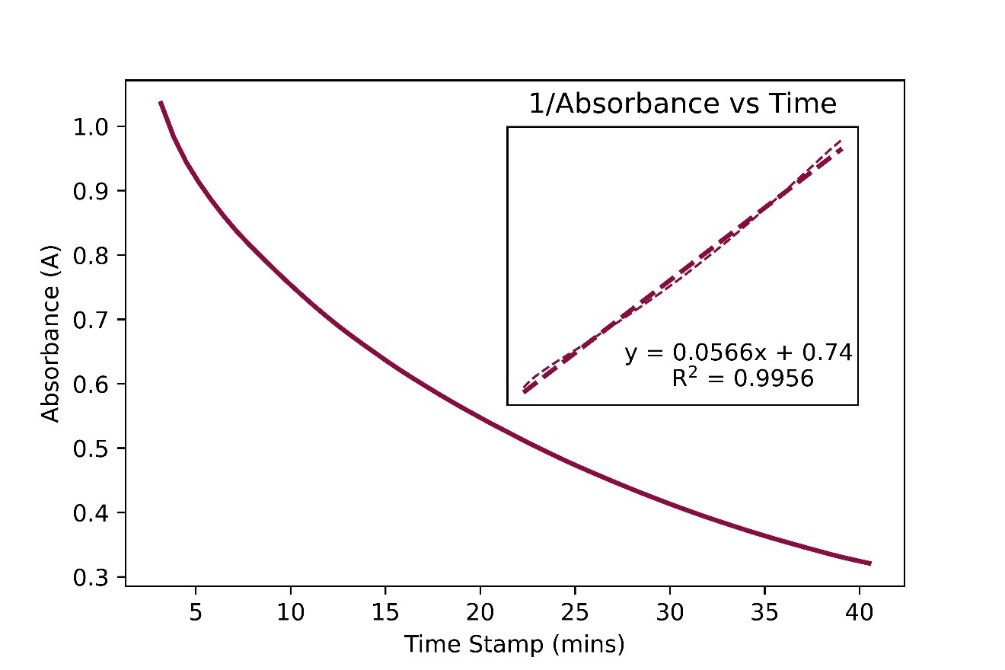
**

Figure S5: Plot showing how A_max_ changes when Cu(DMAP)_2_(OTf)_2_ at 8.5 µmol/L reacts with MN”. Its respective linear trendline when 1/A_max_ is also shown.

**
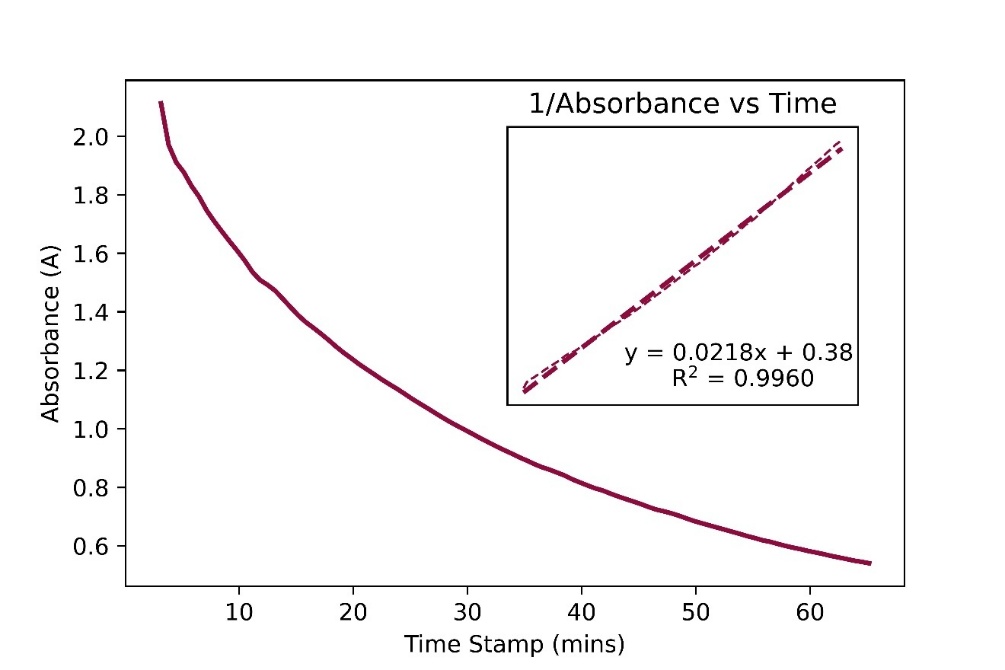
**

Figure S6: Plot showing how A_max_ changes when Cu(DMAP)_2_(OTf)_2_ at 10 µmol/L reacts with MN”. Its respective linear trendline when 1/A_max_ is also shown.


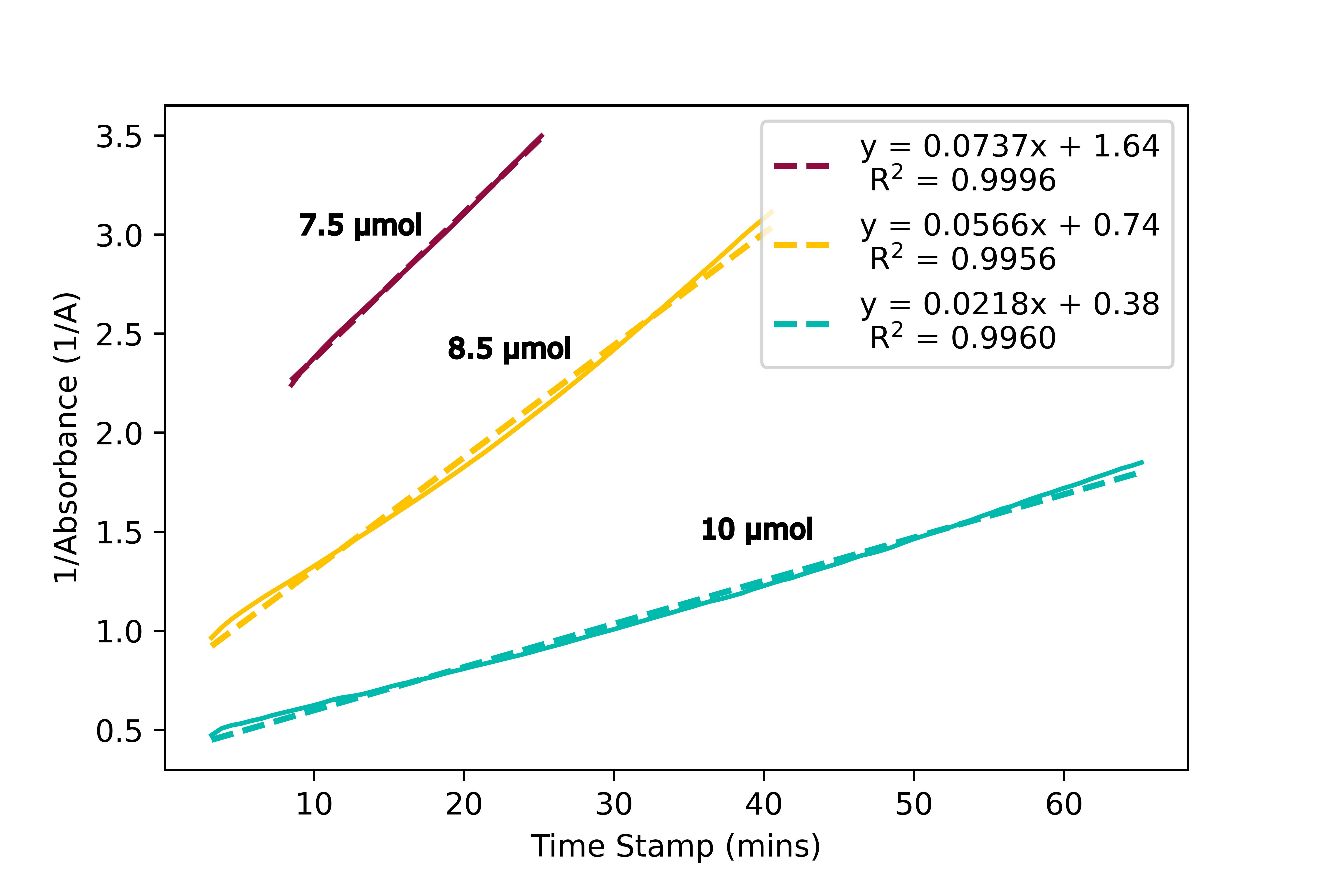
Figure S7: Graph of 1/Max Absorbance (A^−1^ ) vs time (min) showing how the maximum absorbance gathered for reactions of Cu(DMAP)_2_(OTf)_2_ at 7.5, 8.5 and 10 µmol/L with MN” and their respective linear trendlines.

## Kinetic UV/Vis Analysis & Rate Equations

Scheme S4: Proposed mechanism of (DMAP)CuN"2 degradation.

For N” = N(SiMe_3_). The rate of formation of the Li[CuN”_3_] species can be expressed as:

$$\frac{d[Li\{Cu{(N")}_{3}\}]}{dt}=k_{2}\left[ \left( DMAP \right)Cu{(N")}_{2} \right][LiN"] -k_{-2}\left[ Li\{Cu{(N")}_{3}\} \right][DMAP]- k_{3}\left[ Li\{Cu{(N")}_{3}\} \right]$$

We first assume that Li[CuN”_3_] only exists transiently and thus, a steady state approximation can be made and therefore, the above can be written as:

$$\frac{d[Li\{Cu{(N")}_{3}\}]}{dt}=k_{2}\left[ \left( DMAP \right)Cu{(N")}_{2} \right][LiN"] -k_{-2}\left[ Li\{Cu{(N")}_{3}\} \right][DMAP]- k_{3}\left[ Li\{Cu{(N")}_{3}\} \right]\cong0$$

$$k_{2}\left[ \left( DMAP \right)Cu{(N")}_{2} \right][LiN"] =k_{-2}\left[ Li\{Cu{(N")}_{3}\} \right][DMAP]+ k_{3}\left[ Li\{Cu{(N")}_{3}\} \right]$$

$$\left[ Li\{Cu{(N")}_{3}\} \right]= \frac{k_{2}\left[ \left( DMAP \right)Cu{(N")}_{2} \right][LiN"]}{k_{3}+ k_{-2}\left[ DMAP \right]}$$

Assuming that the rate of formation of Cu^I^ occurs from unimolecular degradation of [CuN”_3_]^–^ then:

$$\frac{d[{Cu}^{I}{(N")}]}{dt}= k_{3}\left[ Li\{Cu{(N")}_{3}\} \right]=k_{3}\left( \frac{k_{2}\left[ \left( DMAP \right)Cu{(N")}_{2} \right][LiN"]}{k_{3}+ k_{-2}\left[ DMAP \right]} \right)$$

$$\frac{d[{Cu}^{I}{(N")}]}{dt}\propto k'\left( \frac{\left[ \left( DMAP \right)Cu{(N")}_{2} \right][LiN"]}{\left[ DMAP \right]} \right)$$

For our kinetic experiments, which were carried out with 20-fold excess of DMAP, we assume DMAP is present in a steady state over the course of each run; thus, the observed reaction order of 2 is consistent with these equations. Hence:

$$\frac{d[{Cu}^{I}{(N")}]}{dt}= k_{3}\left[ Li\{Cu{(N")}_{3}\} \right]=k_{3}\left( \frac{k_{2}\left[ \left( DMAP \right)Cu{(N")}_{2} \right][LiN"]}{k_{3}+ k_{-2}\left[ DMAP \right]} \right)$$

$$\frac{d[{Cu}^{I}{(N")}]}{dt}\propto k'\left( \frac{\left[ \left( DMAP \right)Cu{(N")}_{2} \right][LiN"]}{\left[ DMAP \right]} \right)$$

Reactions at three different reagent concentrations (see Figures S3a-c, S4) revealed that the degradation rate was slower at higher concentration than at lower ones, suggesting a high reaction order in DMAP. Modelling the expected rates at different reaction orders w.r.t. DMAP provided Charts S1 & S2.

**
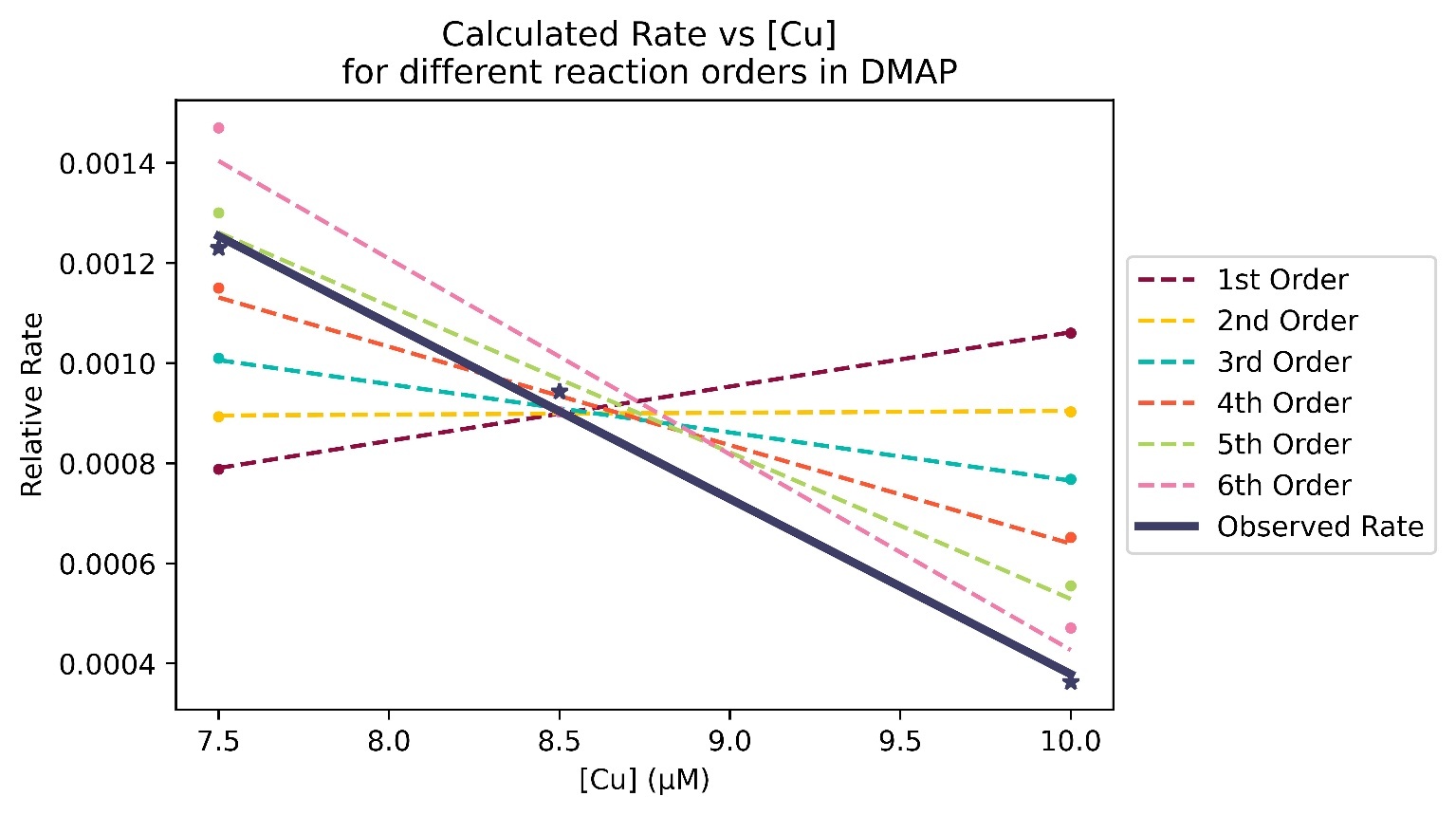
**

Chart S1: Plot of the modelled rate of the reaction versus [Cu] for different negative orders of DMAP alongside the observed rate. Since the observed rate is inversely proportional to [Cu] concentration the reaction order in DMAP must be < –2.

**
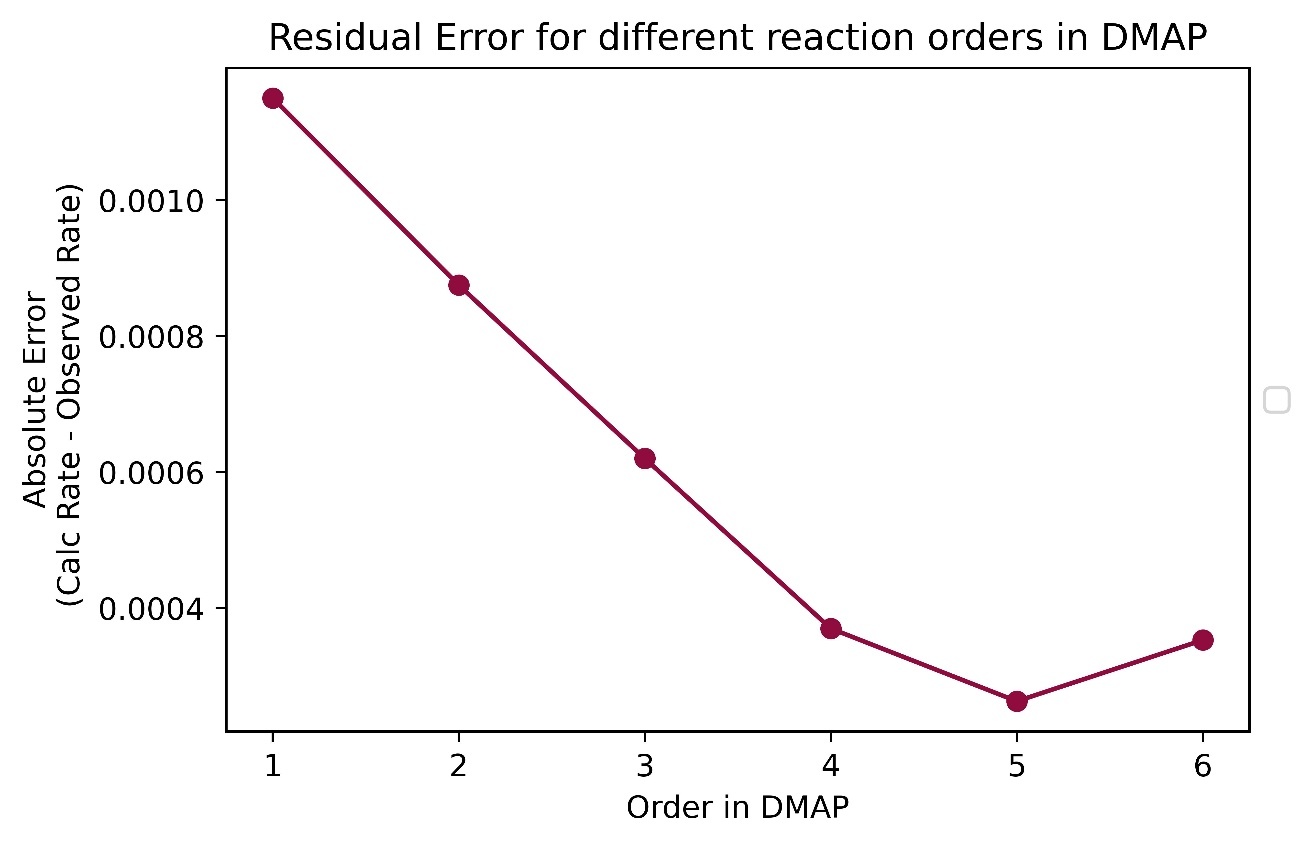
**

Chart S2: Plot of residual errors between the calculated rate and the observed rate determines the reaction order of –5 in DMAP.

The above modelling yielded a negative 5-fold dependence of the rate on DMAP concentration.

(Eq. S1)

$$\frac{d[{Cu}^{I}{(N")}]}{dt}\propto k'\left( \frac{\left[ \left( DMAP \right)Cu{(N")}_{2} \right][LiN"]}{\left[ DMAP \right]^{5}} \right)$$

This may suggest that the formation of a DMAP solvated Li ion (i.e. Li(DMAP)_4_) is key to stabilizing the proposed Cu^I^N”_3_ anion towards reduction.

## Optimization Table (Table 1) Protocol

To a suspension of CuOTf_2_(DMAP)_n_ (n = 2, 3, 4) in the solvent (ca 0.05 M) was added a solution of 1,3,5-tri-tert-butylbenzene in the solvent (1.8 equiv.) followed by a solution of the hexamethyldisilazide salt in the solvent (1.8 equiv.). The resulting solution (20 mM in Cu) was stirred for 90 minutes before the solution was separated from the salts by either filtration or centrifugation. To the mother liquor was added a few drops of C_6_D_6_ and the solution was analysed by ^1^H NMR spectroscopy. Automated addition of the reagent was achieved using a LSPOne syringe pump/valve system as detailed above. Dropping funnel addition was achieved using a 3-necked 100 mL RBF flask appended with a 50mL dropping funnel and attached to a Schlenk line.

## NMR Spectra

### **
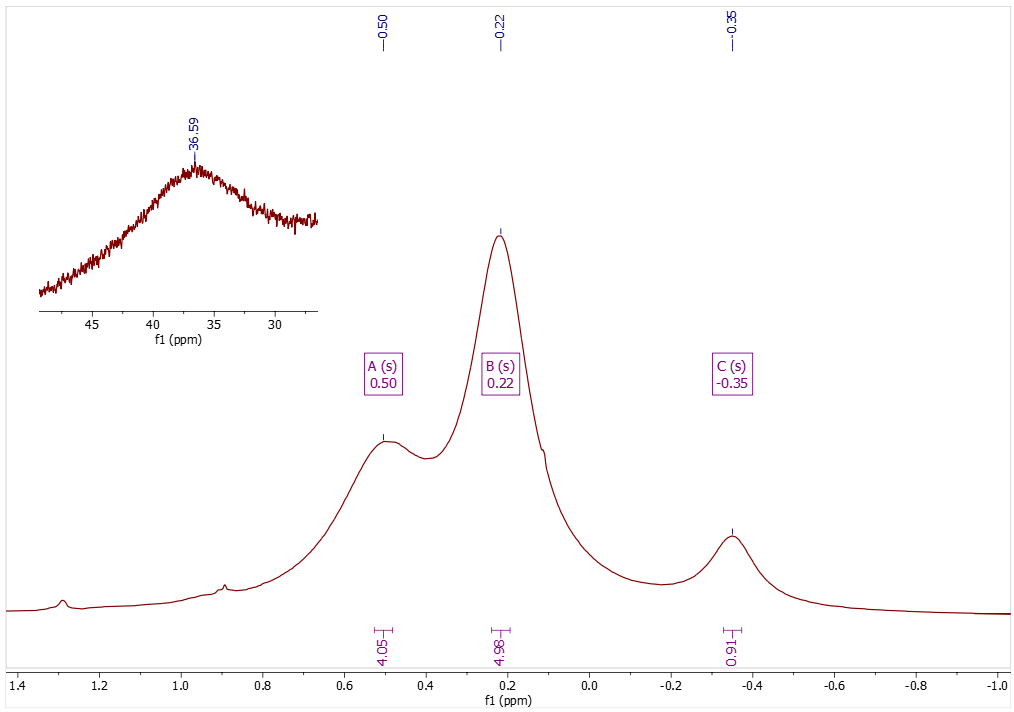
**H^1^ NMR Spectrum of [(DMAP)_2_Cu(OTf)_2_] at 25 °C.

Figure S8: ^1^H NMR spectrum of [(DMAP)_2_Cu(OTf)_2_] in region -1 to 1.5 ppm at 25 °C showing N(CH_3_)_2_ resonances with inset showing CH_Ar_ at 37 ppm. No other resonances for the aromatic protons are observed due to paramagnetism.

### **
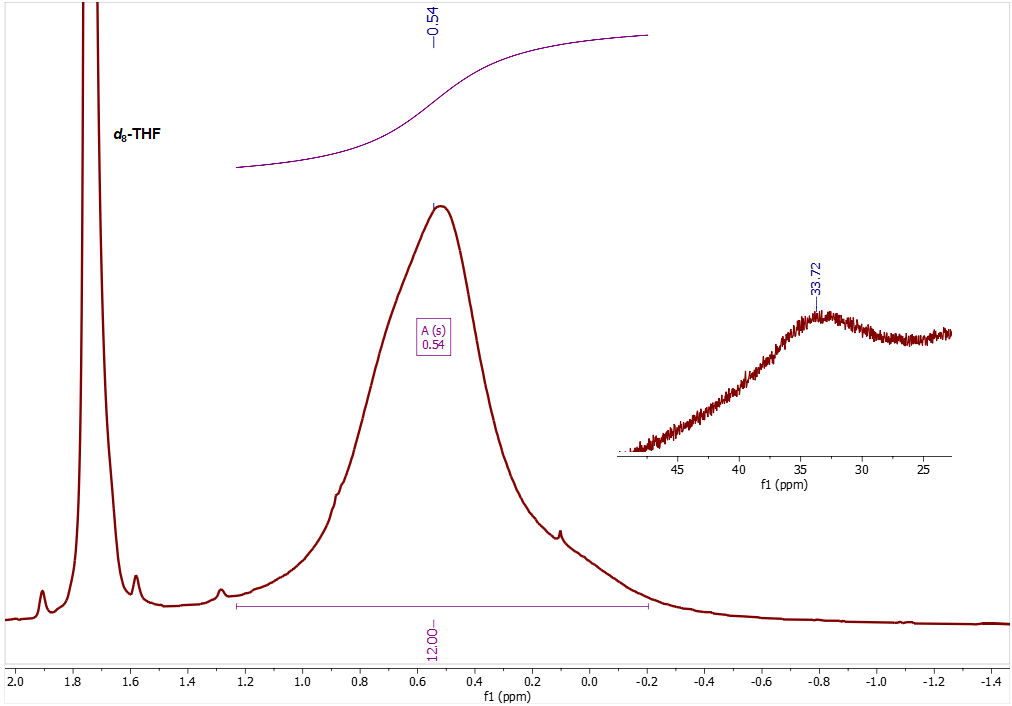
**H^1^ NMR Spectrum of [(DMAP)_2_Cu(OTf)_2_] at 50 °C

Figure S9: ^1^H NMR spectrum of [(DMAP)_2_Cu(OTf)_2_] in region -1.5 to 2 ppm at 50 °C showing coalesence into a sigle N(CH_3_)_2_ resonance with inset showing CHAr at 34 ppm. No other resonances for the aromatic protons are observed due to paramagnetism.

### ^1^H NMR Spectrum of [(DMAP)_4_CuOTf_2_]

**
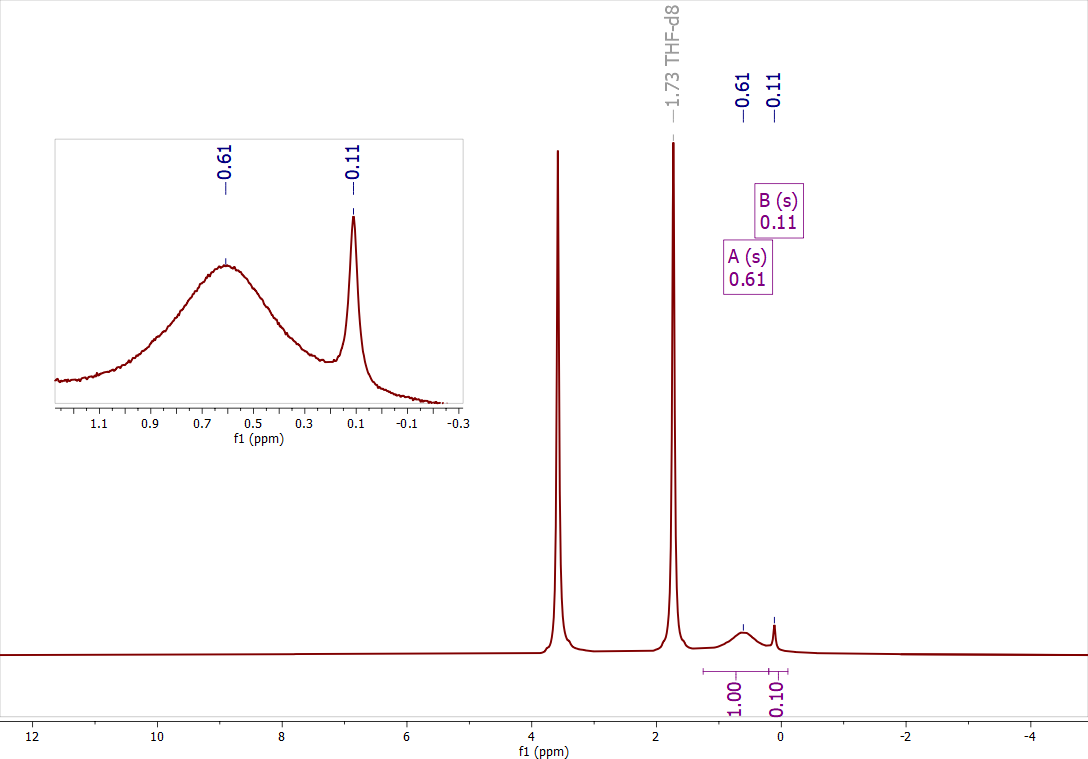
**

Figure S10: ^1^H NMR spectrum of [(DMAP)_4_Cu(OTf)_2_] at 25°C in *d_8_*-THF.

### ^1^H NMR Spectrum of [(DMAP)Cu{N(SiMe_3_)_2_}_2_]

**
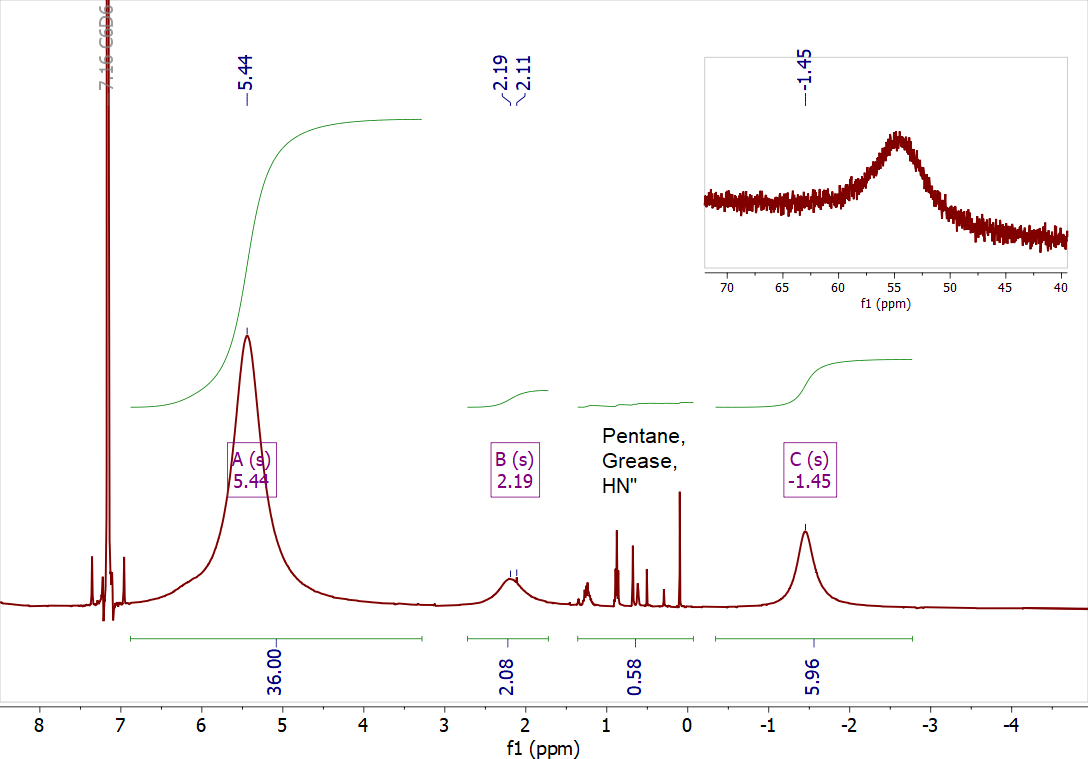
**

Figure S11: ^1^H NMR Spectrum of isolated [(DMAP)Cu{N(SiMe_3_)_2_}_2_] in C_6_D_6_.

## EPR Spectra


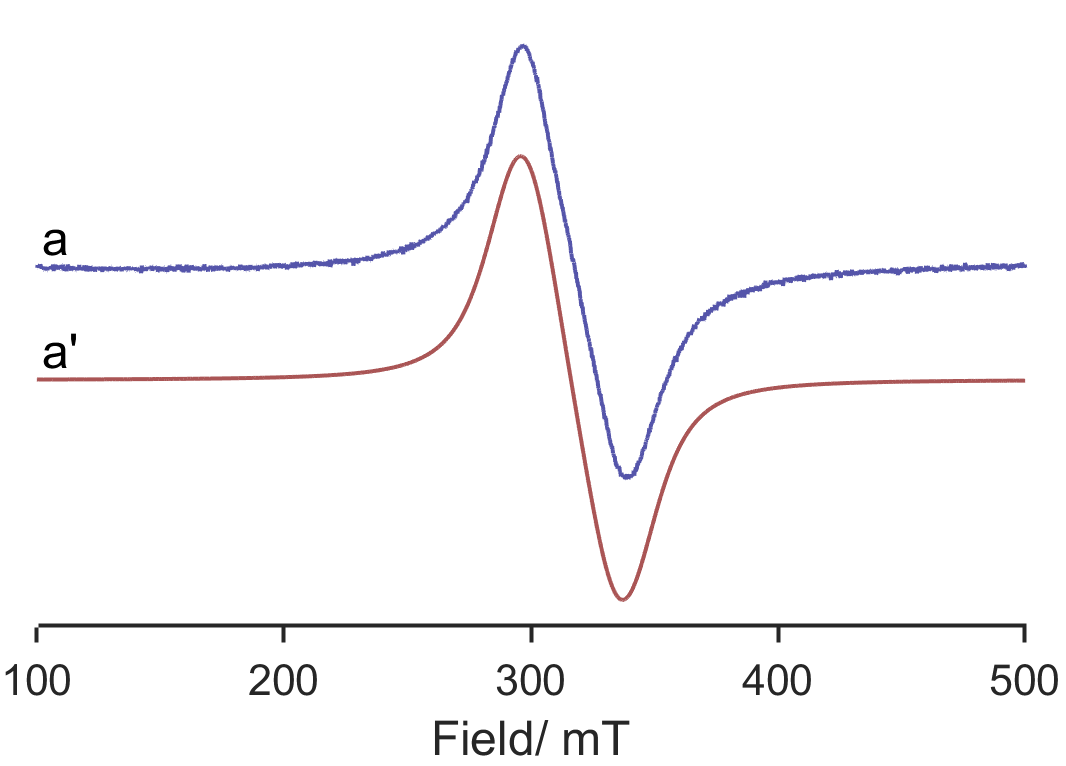


**Figure S12** CW X-band EPR spectrum (T = 298 K) of [(DMAP)Cu(N(SiMe_3_)_2_] in toluene:fluorobenzene (a) experiment, (a′) simulation.


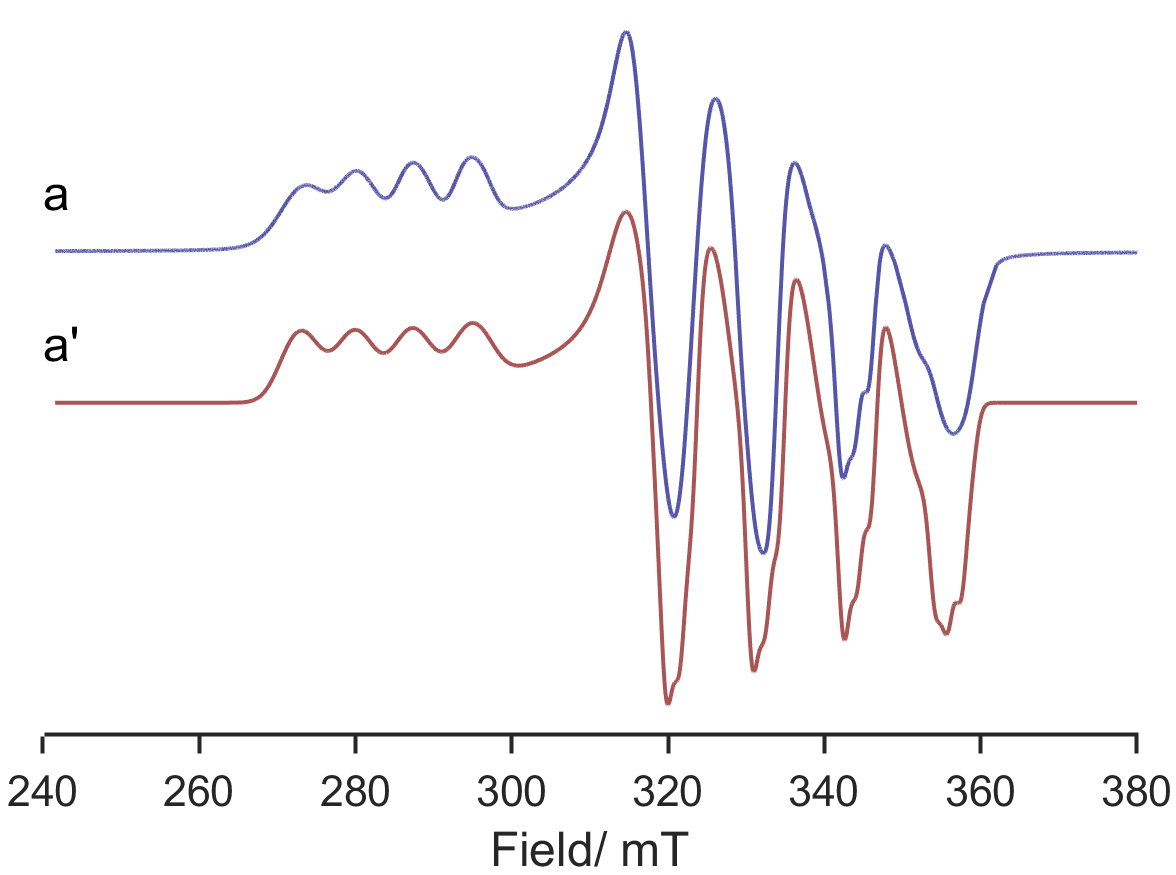


**Figure S13** CW X-band EPR spectrum (T = 120 K) of [(DMAP)Cu(N(SiMe_3_)_2_] in toluene:fluorobenzene (a) experiment, (a′) simulation.

The frozen solution EPR spectrum of [(DMAP)Cu(N(SiMe_3_)_2_] in a toluene: fluorobenzene (2:1) solvent system revealed a predominantly axial signal with clearly resolvable copper hyperfine coupling. Upon close inspection, some features attributable to nitrogen superhyperfine coupling can also be weakly resolved on one component at ~ 340 mT.

A satisfactory simulation of the signal was achieved using a slightly rhombic **g**-tensor, and the inclusion of one nitrogen nucleus (see details below) suggesting that the origin of this coupling is attributed to coordination of the DMAP pyridine nitrogen.

The room temperature spectrum could be fully simulated by sole inclusion of the averaged isotropic values (Tablse S2) of the (DMAP)Cu^II^(N{SiMe_3_}_2_)_2_ centre, notably there was no evidence of an organic radical based signal which could be attributed to a Cu(I)-complex with radical ligand.

**Table S2**: Spin Hamiltonian parameters of [(DMAP)Cu(N(SiMe_3_)_2_] *^i^*

|  | *g*_1_ | *g*_2_ | *g*_3_ | *g*_iso_ |
| --- | --- | --- | --- | --- |
|  | 2.0055  ± 0.0005 | 2.0360  ± 0.005 | 2.3995  ± 0.005 | 2.147 |
|  |  |  |  |  |
|  | **A_1_**/ MHz | **A_2_**/ MHz | **A_3_**/ MHz | **a_iso_**/ MHz |
| ^63^Cu | 323  ± 10 | 299  ± 10 | 239  ± 5 | 287 |
| ^14^N | 48  ± 5 | 40  ± 5 | 54  ± 5 | 47 |

*^i^* The **g**-tensor and **A**-tensors are assumed to be co-linear (i.e. Euler angles α,β,γ = 0).

## Computational Modeling of DMAPCuN"_2_

Geometry optimisations and subsequent UV-Vis predictions were performed using Gaussian09^[3]^ utilizing the PBE1PBE level of theory with a Def2SVP basis sets applied to all atoms and and Grimme's empirical dispersion correction with Becke-Johnson Damping (D3-BJ). EPR parameters were computed on the M062X level of theory with Def2TZVP basis sets.^[4]^ No solvent corrections were applied. All optimised molecular structures where checked to be minima on the energy hypersurface and possess no imaginary vibrational frequencies. Natural Bond Orbital Theory was applied to study the electronic states.The procedure for computational calculations was taken from Kaiser *et. al.* to enable comparison between various Cu species.

Spin density on Cu calculated to be 0.606453 (PBE1PBE/Def2SVP) and 0.607121 (M062X/Def2TZVP).

## Geometry Optimisation Data for DMAPCuN"_2_

Computational methodology for DMAPCuN"_2_ was validated by running energy optimisations for the complex Cu^II^N"_2_ computational details for which were reported by Kaiser *et. al.^[4]^*

Table S3: Spin Density calculations for **2** and comparable examples (PBE1PBE/Def2SVP).

|  | **E with dispersion/ a.u.** | **Spin Density Cu** | **Spin Density N** |
| --- | --- | --- | --- |
| (R^+•^)Cu^I^OTf^[4]^ | -4518.320150 | 0.146 | 0.362 |
| Cu^II^(N{Si*^i^*Pr_3_}Dipp)_2_^[4]^ | -3970.219726 | 0.228 | 0.356 |
| Cu^II^N"_2_^[4]^ | -3384.3846060 | 0.305 | 0.318 |
| Cu^II^N"_2_^†^ | -3384.8017920 | 0.320 | 0.314 |
| **DMAPCu^II^N"_2_ (**2**)^†^** | **-3766.394937** | **0.606** | **0.130 (N”)** |

^†^This Work

Table S4: Comparison of experimental and calculated geometries (PBE1PBE/Def2SVP)

| **DMAPCuN"_2_** | |
| --- | --- |
| **Calculated Geometry** | **Experimental Geometry** |
| 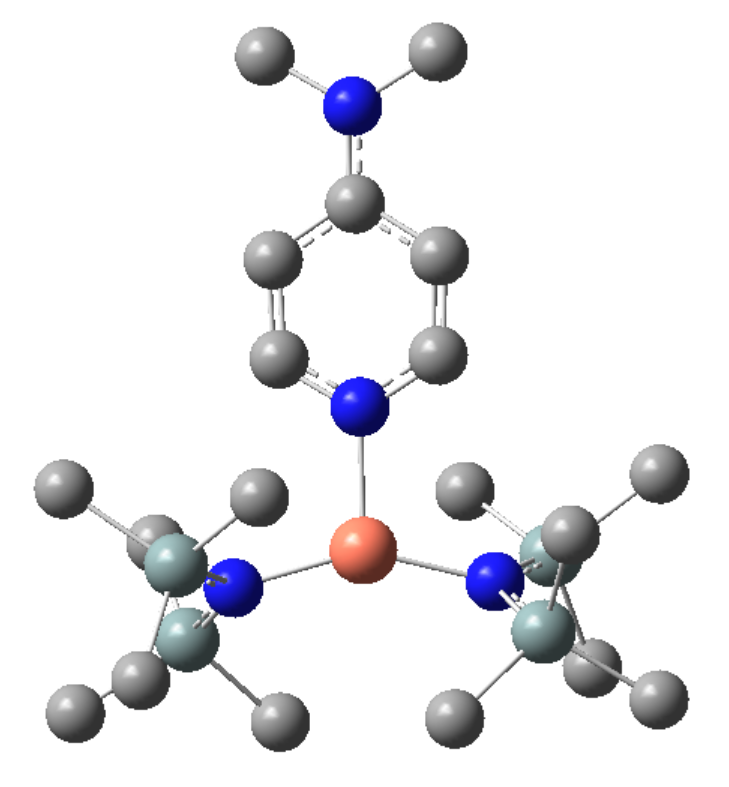  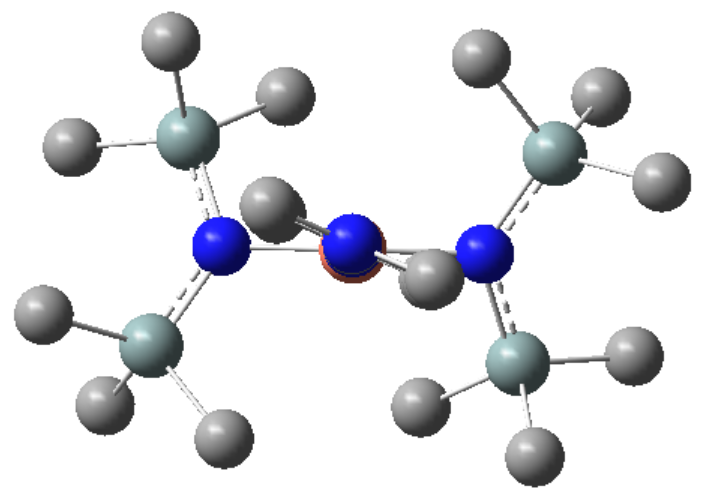 | 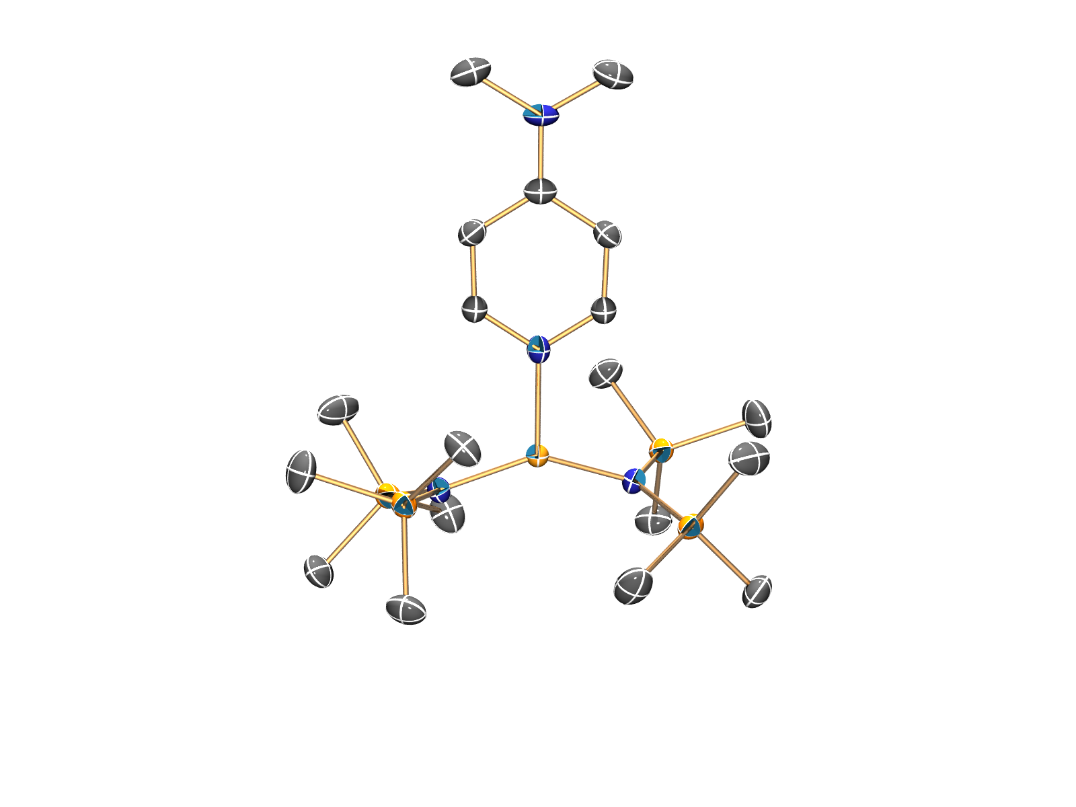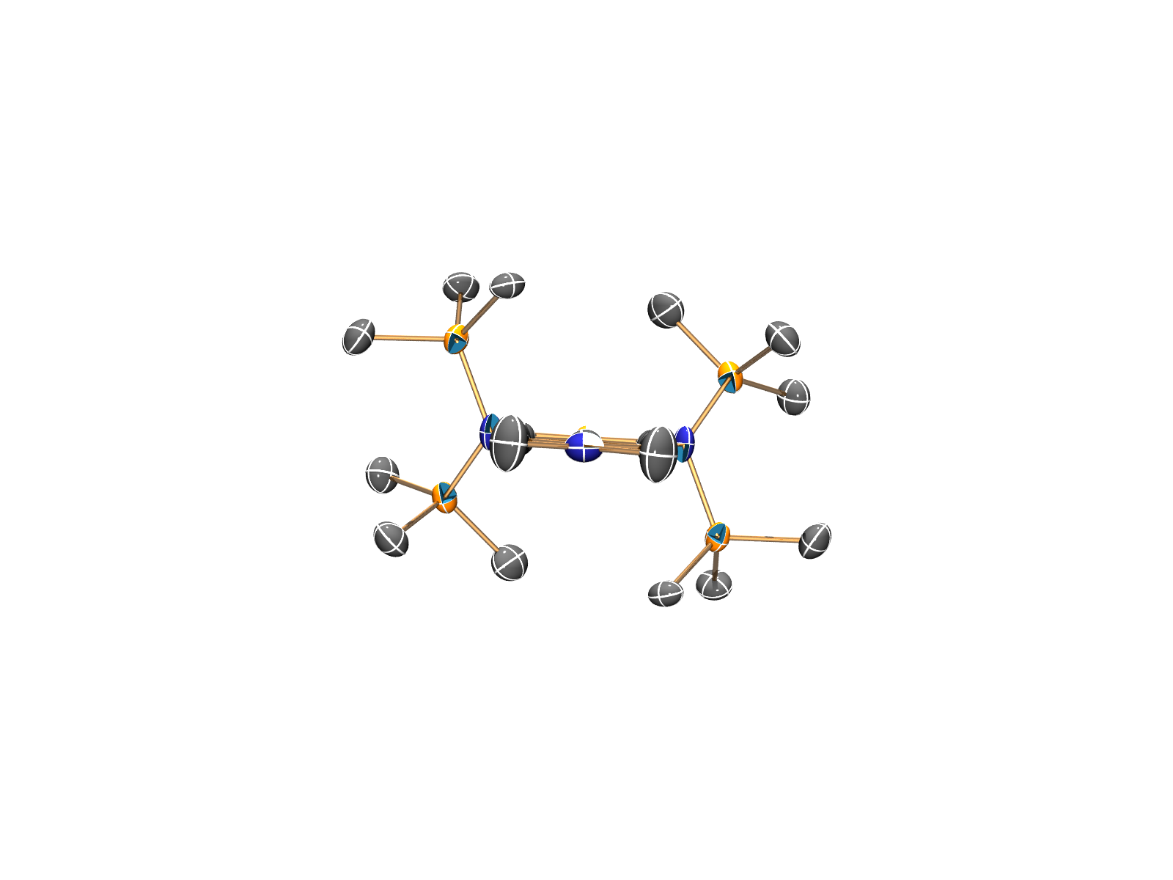 |
|  |  |

Table S5:Showing the experimental and computational structural details like bond lengths and bond angles. Bond lengths are listed as averages for the same bond type.

|  | **Experimental** | **PBE1PBE/Def2SVP** |
| --- | --- | --- |
| Initial Multiplicity Used | - | 2 |
| Calced. Multiplicity | - | 2 |
| Mulliken Charge on Cu | - | 0.488 |
| Spin Density on Cu | - | 0.606 |
| Cu-N_DMAP_ (Å) | 1.952(3) | 1.996 |
| Cu-N_SiMe3_(Å) | 1.8819(18) | 1.886 |
| N-Si (Å) | 1.7170(18)/1.7139(18) | 1.731 |
| (_ortho_C-N_endo_)_DMAP_ (Å) | 1.357(3) | 1.340 |
| (C_ortho_-C_meta_)_DMAP_ (Å) | 1.368(3) | 1.380 |
| (C_meta_-C_para_)_DMAP_ (Å) | 1.416(3) | 1.417 |
| (N_exo_-C_Me_)_DMAP_ (Å) | 1.444(3) | 1.444 |
| (N_exo_-C_para_)_DMAP_ (Å) | 1.357(3) | 1.356 |
| N_SiMe3_-Cu-N_DMAP_ (^o^) | 107.35(6) | 104.6 |


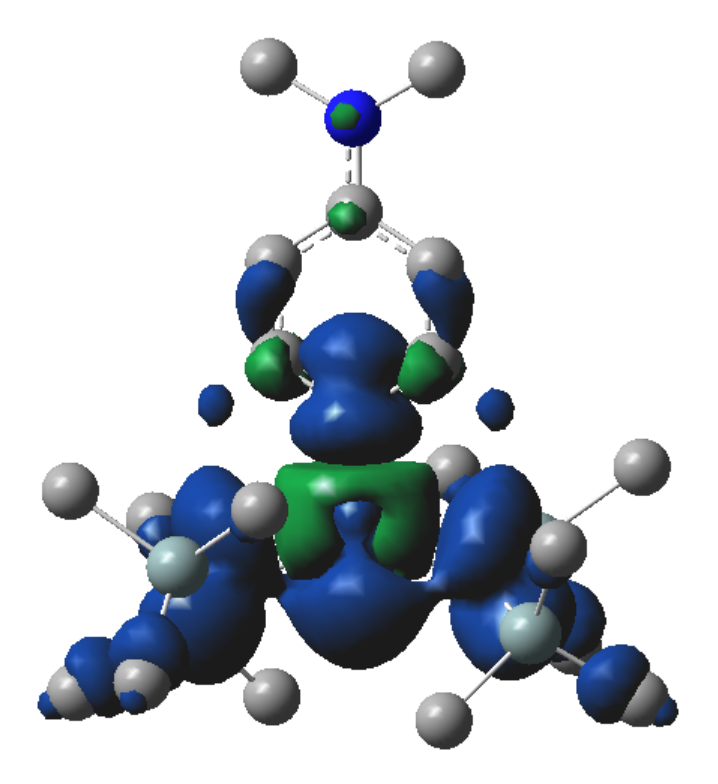

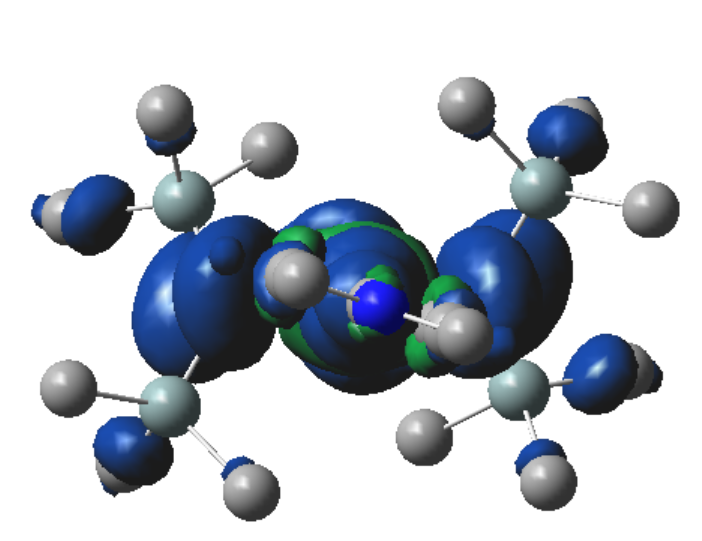

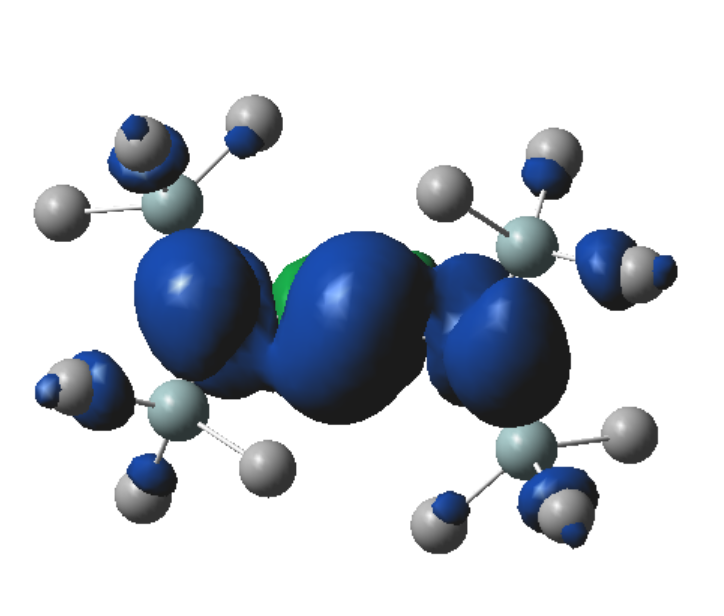


**Figure S14**: Spin density plots of [(DMAP)Cu{N(SiMe_3_)_2_}_2_] with isosurfaces set to 0.02.

## _
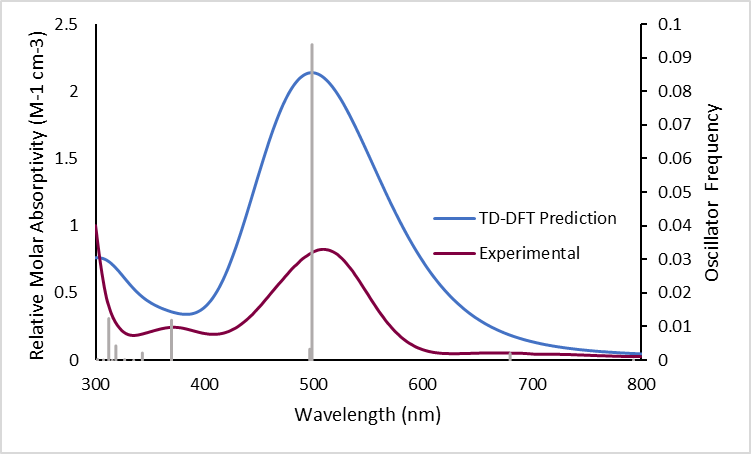
_Details for TD-DFT Calculations on DMAPCuN"_2_

**Figure S15**:Plot showing the experimentally obtained molar absorbance vs wavelength graph of [(DMAP)Cu{N(SiMe_3_)_2_}_2_] (1.8 mM) in THF (red) and the TD-TDF predicted spectrum for the same species (blue) as well as the oscillator frequencies of possible transitions from 300-800 nm. The predicted spectrum is shown for the SOMO-3 🡪 SOMO (134β -> 137 β) transition. Molar absorbance values were scaled relative to the highest value.


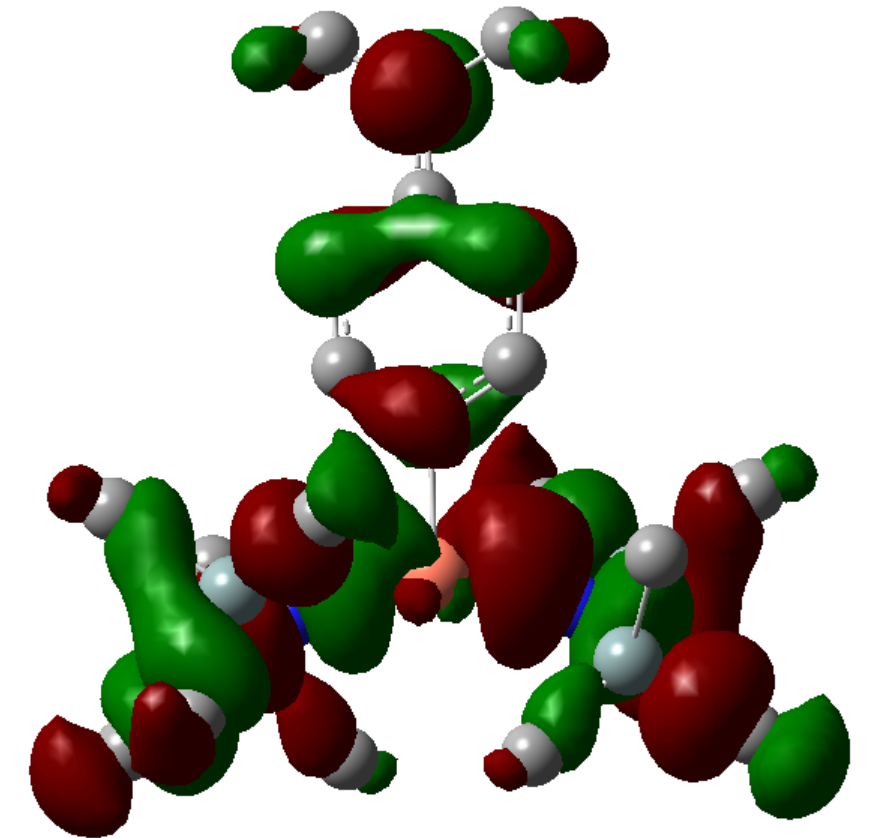


SOMO-3 134B


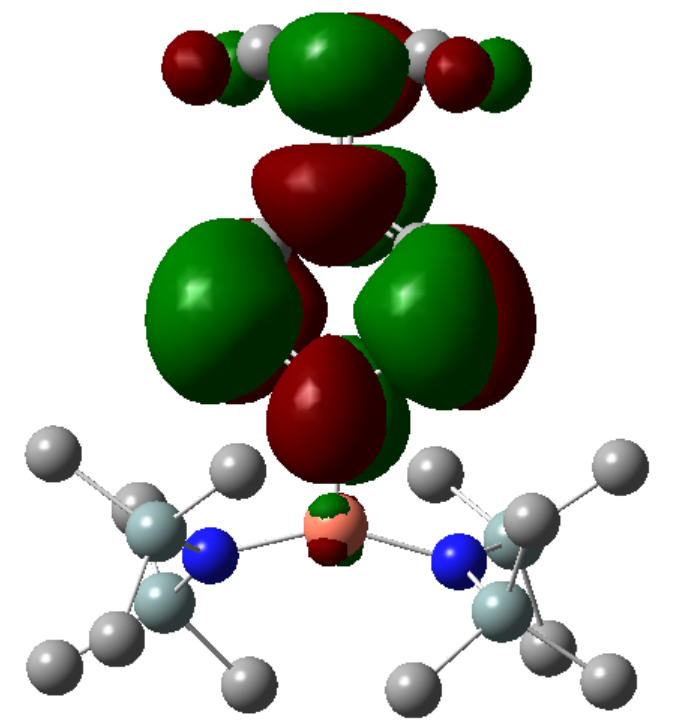


138B LUMO


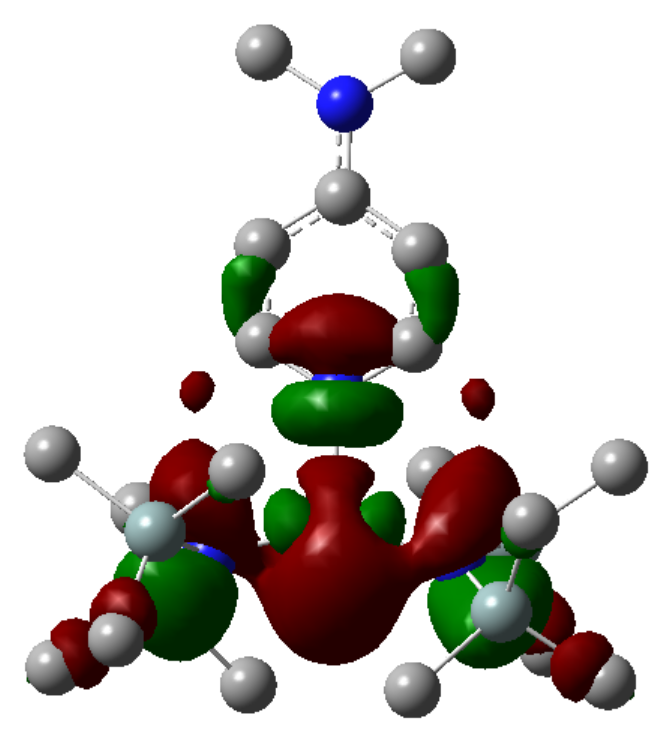


137B SOMO

(unoccupied)


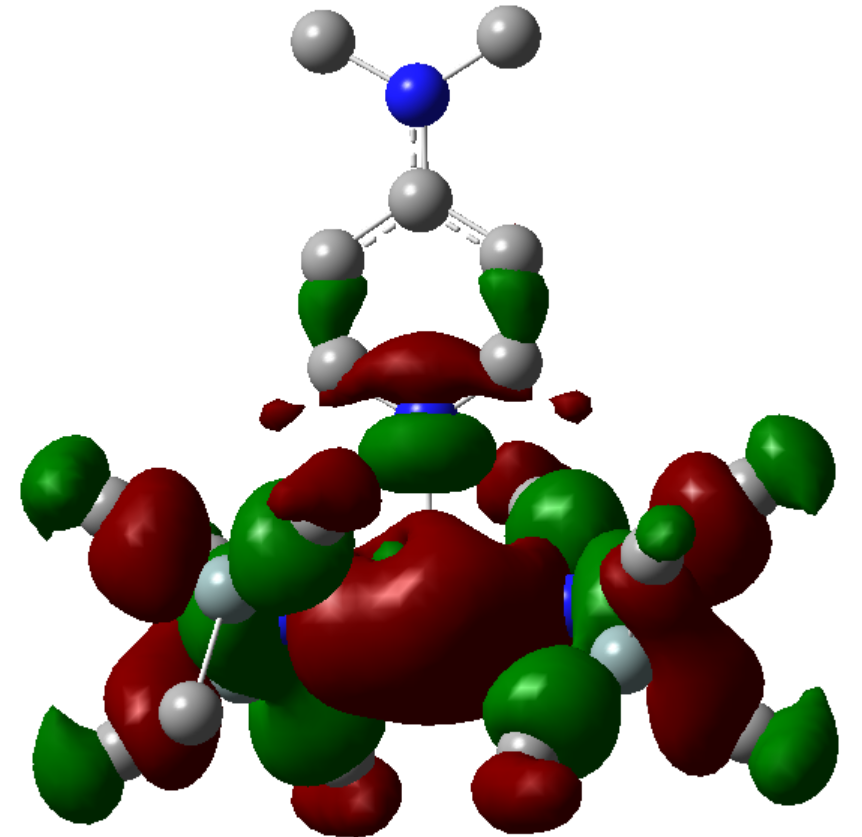


SOMO-3 134A


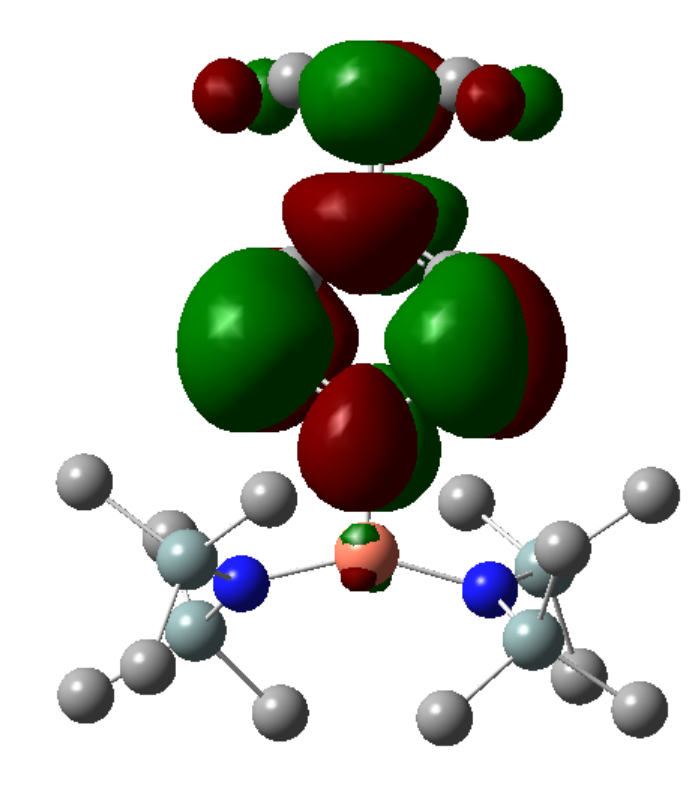


138A LUMO


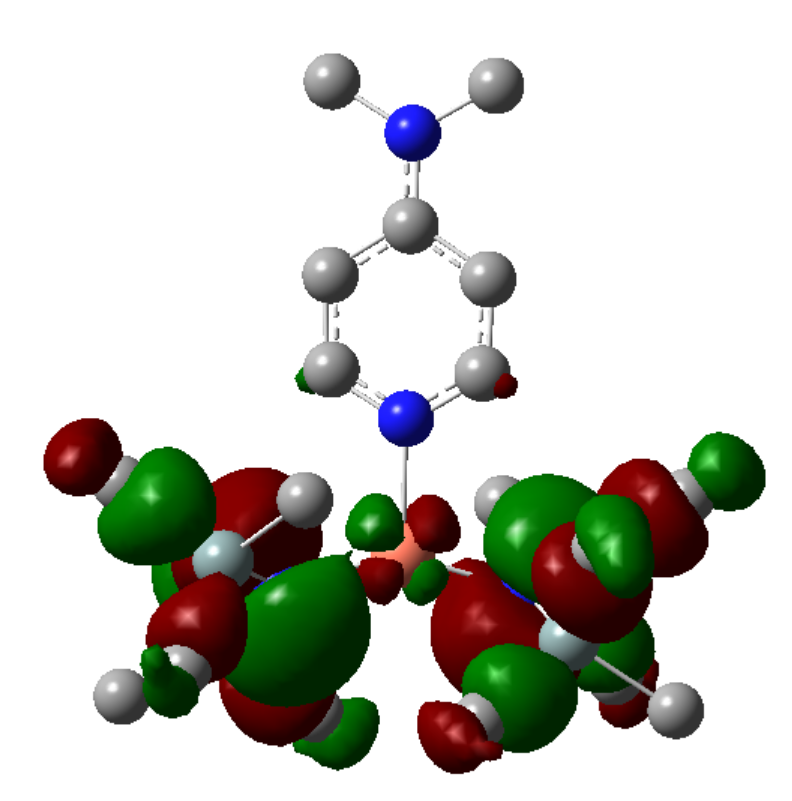


137A SOMO

-11.38 eV

-11.55 eV

-11.61 eV

-11.59 eV

-11.90 eV

-12.12 eV

Excited State 5: 2.014-A 2.4880 eV 498.33 nm f=0.0939 <S**2>=0.764

106B -> 137B -0.11879

113B -> 137B -0.24234

123B -> 137B -0.20630

125B -> 137B -0.16780

133B -> 137B -0.15778

**134B -> 137B 0.72268**

136B -> 137B -0.51787

**Optimised Coordinates of DMAPCuN"_2_**

0 2

Si 1.85348200 2.30410400 1.54379300

Si 0.80776900 2.78924700 -1.35827900

N 1.14528200 1.82479200 0.03730900

C 0.51475000 2.55184100 2.85934300

H -0.17818500 3.35680500 2.56317500

H 0.94848800 2.82493200 3.83506000

H -0.07702300 1.63306700 3.00463000

C 2.83724600 3.90928400 1.41631700

H 3.69213600 3.79560700 0.73201200

H 3.23299900 4.18697000 2.40670100

H 2.22165700 4.74813700 1.05567800

C 3.02965900 0.95858200 2.14994300

H 2.53198500 -0.02165400 2.22300800

H 3.44933800 1.19832800 3.14023700

H 3.86446500 0.84269700 1.44020200

C -0.28624300 4.28231200 -0.95877700

H -1.26979600 3.96304800 -0.57758200

H -0.46042500 4.90286600 -1.85297100

H 0.17621200 4.92437200 -0.19228700

C 2.38765300 3.42298400 -2.17238200

H 2.93626900 4.11070000 -1.51141400

H 2.16325600 3.96017100 -3.10831800

H 3.05722800 2.58166400 -2.41189200

C -0.10840000 1.76147900 -2.65564900

H 0.46168200 0.86342500 -2.94390400

H -0.25526100 2.36094400 -3.56884300

H -1.09987400 1.43525600 -2.30678700

C -2.01340900 -1.05814200 -0.44910700

H -1.41050500 -1.89955000 -0.79706400

C -3.39250200 -1.10472400 -0.47318900

H -3.87526900 -2.00131400 -0.85762700

C -6.22089400 -1.14948500 -0.49187300

H -5.99284400 -1.35510300 -1.55181100

H -7.29774400 -0.96249100 -0.41030600

H -5.99069900 -2.05861400 0.08971100

Cu 0.67078900 -0.00000700 -0.00000400

Si 1.85347000 -2.30415900 -1.54376200

Si 0.80763500 -2.78925600 1.35827300

N 1.14523400 -1.82481800 -0.03730300

N -1.32522200 0.00001300 0.00000100

N -5.49910700 0.00006600 -0.00002800

C 0.51477800 -2.55186400 -2.85935900

H -0.17819600 -3.35680100 -2.56321000

H 0.94854300 -2.82497900 -3.83505800

H -0.07695700 -1.63307000 -3.00467700

C 2.83718400 -3.90936600 -1.41624600

H 3.69205800 -3.79570800 -0.73191900

H 3.23295600 -4.18707500 -2.40661600

H 2.22156000 -4.74819700 -1.05561500

C 3.02970800 -0.95867700 -2.14988200

H 2.53206900 0.02157600 -2.22296500

H 3.44940600 -1.19844000 -3.14016400

H 3.86449900 -0.84281700 -1.44012000

C -0.28641100 -4.28228600 0.95873300

H -1.26993200 -3.96299000 0.57748400

H -0.46066200 -4.90282600 1.85292300

H 0.17606100 -4.92436900 0.19227300

C 2.38746300 -3.42304100 2.17245100

H 2.93608800 -4.11077900 1.51151300

H 2.16300500 -3.96021400 3.10838000

H 3.05705500 -2.58174100 2.41198800

C -0.10855300 -1.76144000 2.65559000

H 0.46155400 -0.86340800 2.94386400

H -0.25548400 -2.36089200 3.56878100

H -1.09999500 -1.43517700 2.30667600

C -2.01338900 1.05818000 0.44911200

H -1.41046800 1.89957300 0.79707500

C -3.39248000 1.10479900 0.47317000

H -3.87523000 2.00140300 0.85759700

C -4.14262100 0.00004800 -0.00001600

C -6.22087200 1.14963600 0.49180300

H -5.99283400 1.35525000 1.55174400

H -7.29772600 0.96267000 0.41021800

H -5.99064500 2.05875800 -0.08977900

## X-ray Diffraction Data


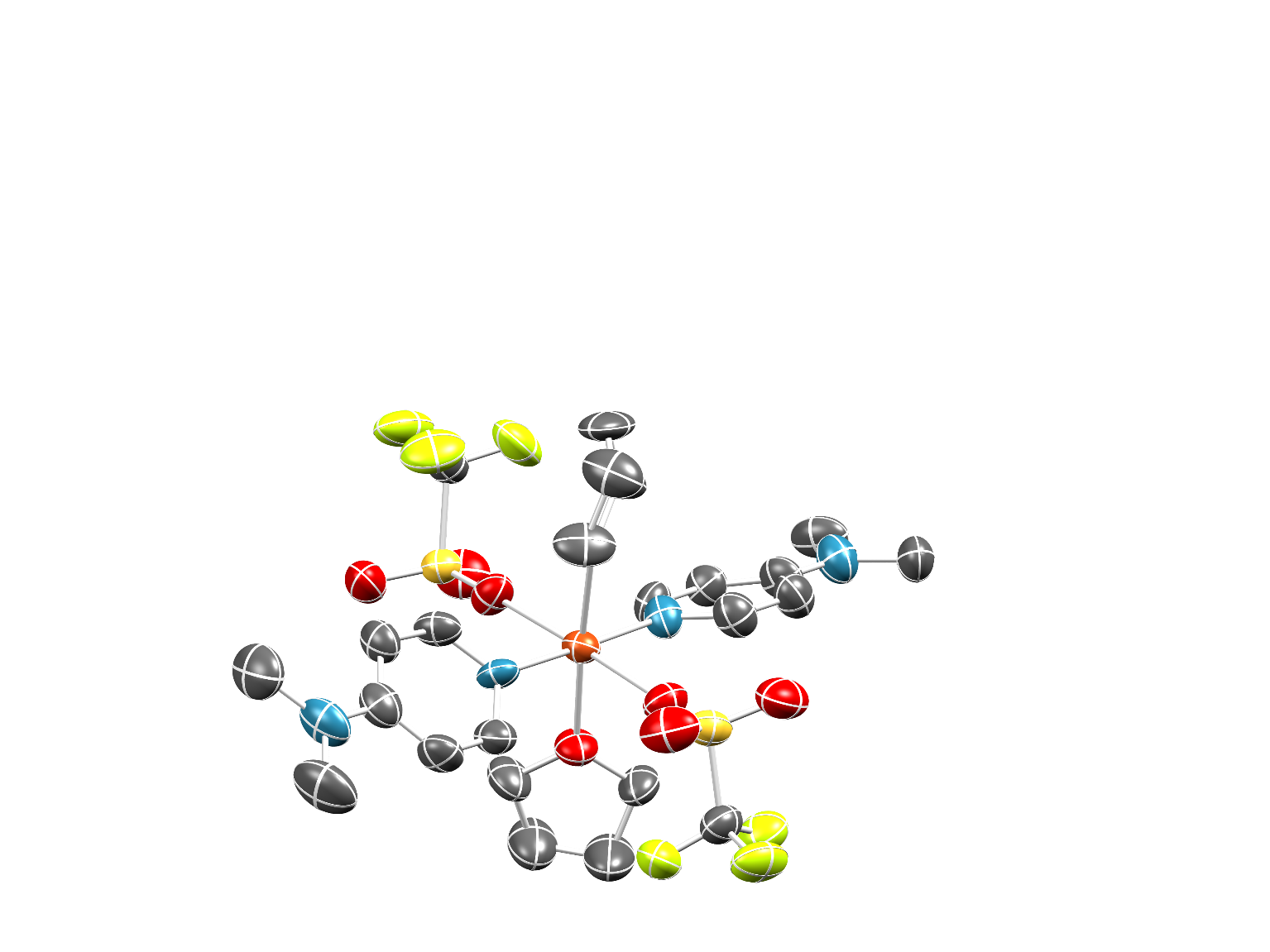


Figure S16: Solid State Structure of the THF solvate of DMAP_2_CuOTf_2_. Thermal ellipsoids set at 50% probability. H-atoms removed for clarity. Only 1/3 of the asymmetric unit is shown. Average geometric parameters across all three moieties in the asymmetric unit are shown in Table S2. Atom colors: Orange, Cu; Red, O; Yellow, S; Green, F; Blue, N, Black, C.


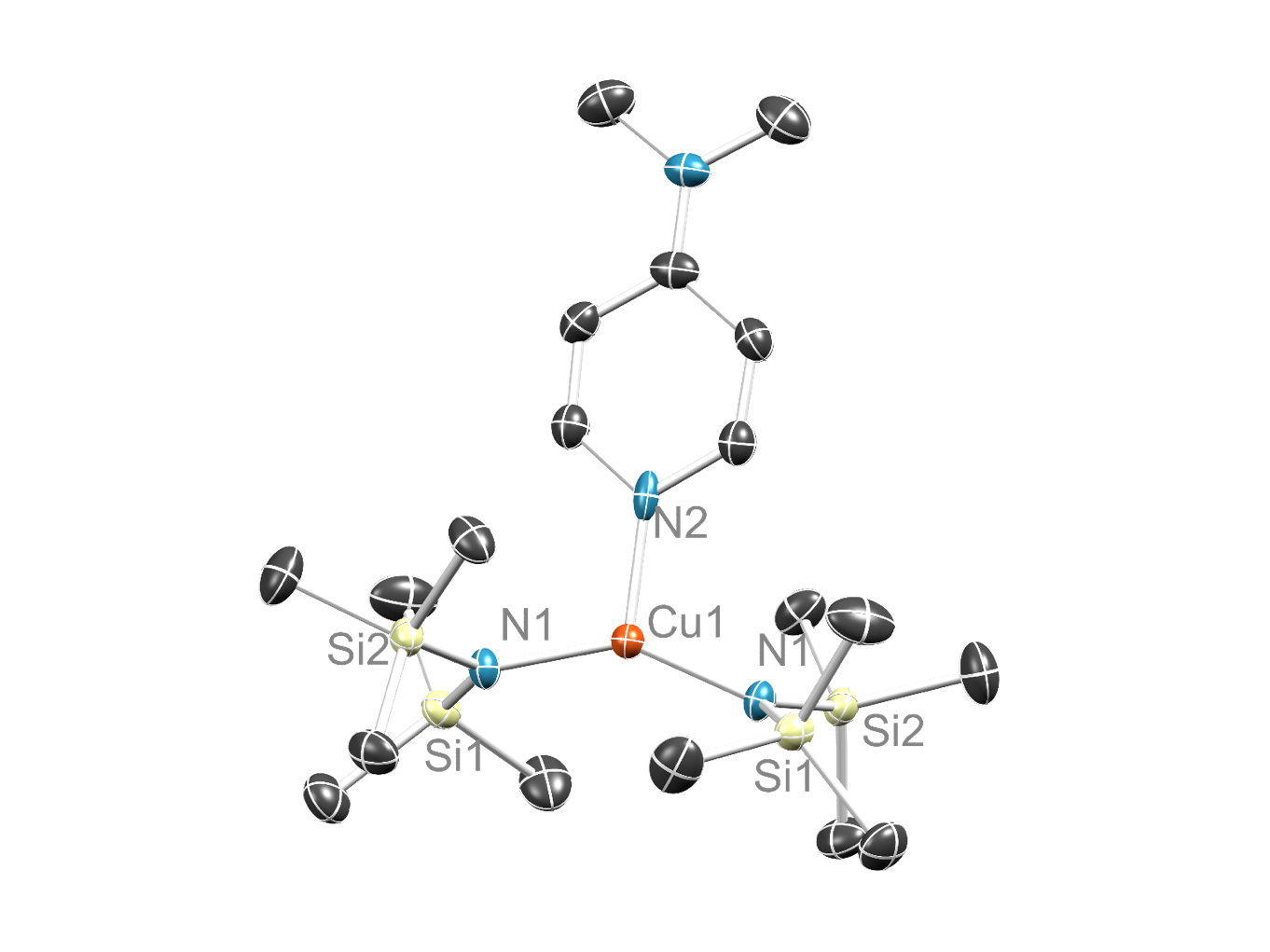


Figure S17: Solid State Structure of DMAPCuN"_2_. Thermal elipsoids set at 50% probability. H-atoms removed for clarity.


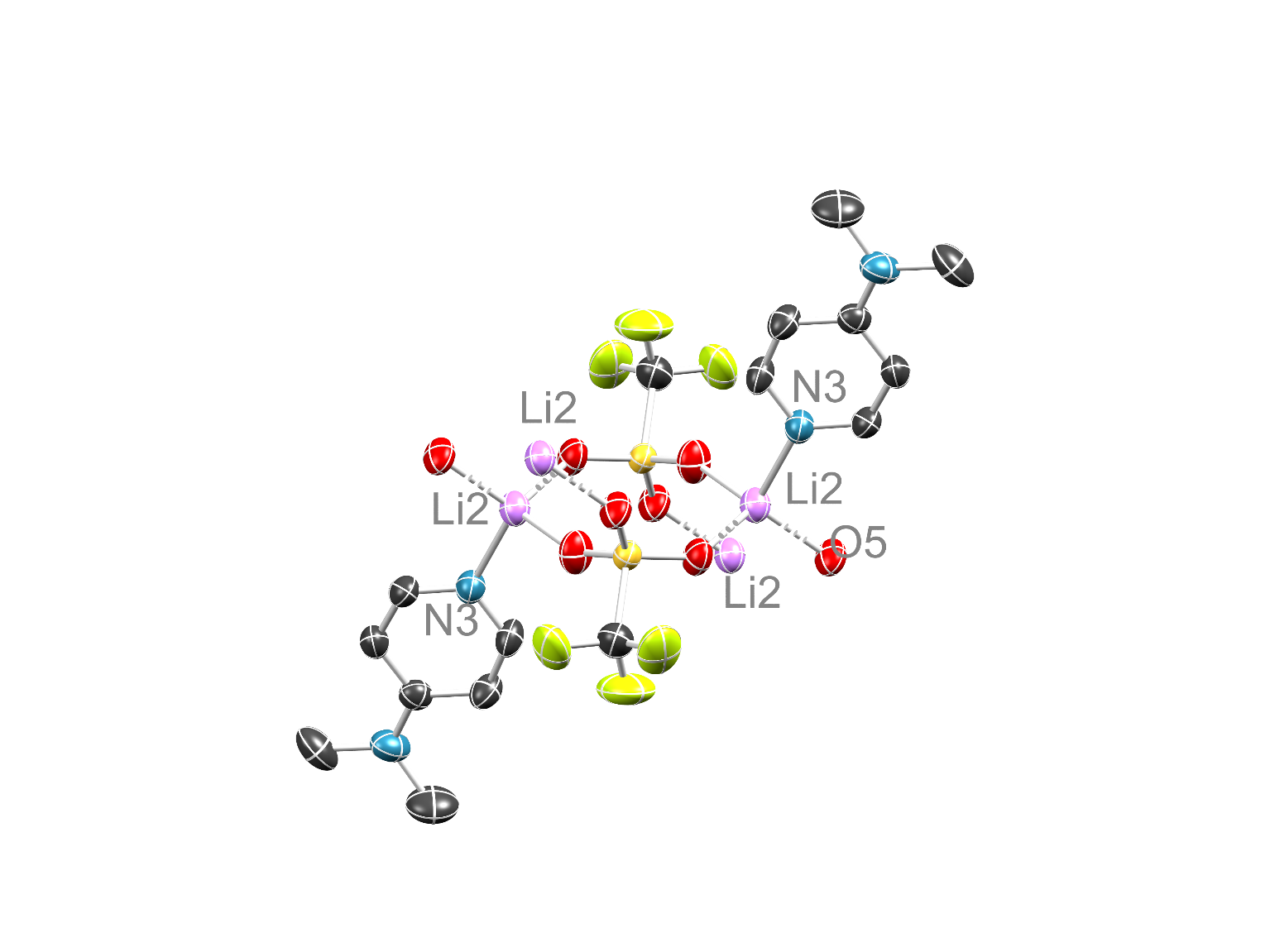
Figure *S*18: Asymmetric unit of the solid*-*state structure of a DMAP.LiOTf by-product. Thermal elipsoids set at 50% probability. H-atoms and lattice C_6_D_6_ removed for clarity.

Table S6: Selected geometric parameters extracted from SCXRD data. Note: Bond lengths and angles for CuDMAP_2_OTf_2_THF_2_ are quoted as averages of the whole asymmetric unit.

| CuDMAP_2_OTf_2_THF_2_ | | | |
| --- | --- | --- | --- |
| Bond Length (Å) | | Bond Angle (º) | |
| Cu—N (DMAP) | 1.97 | N—Cu—N’ (DMAP→DMAP) | 177 |
| Cu—O (THF) | 2.05 | N—Cu—O (DMAP→THF) | 90 |
| Cu—O (OTf) | 2.42 | N—Cu—O (DMAP→OTf) | 90 |
| DMAPCuN"_2_ | | | |
| Bond Length (Å) | | Bond Angle (º) | |
| Cu1—N1 | 1.8819 (18) | N1—Cu1—N2 | 107.35 (6) |
| Cu1—N2 | 1.952 (3) | Si1—N1—Cu1 | 116.82 (9) |
| Si1—N1 | 1.7170 (18) | Si2—N1—Cu1 | 118.10 (10) |
| Si2—N1 | 1.7139 (18) | Si2—N1—Si1 | 124.71 (11) |
| DMAP.LiOTf | | | |
| Bond Length (Å) | | Bond Angle (º) | |
| Li1— N1 | 2.019(5) | C5—N1—Li1 | 127.8 (2) |
| Li2— N3 | 2.023 (4) | C10—N3—Li2 | 125.2 (2) |
| Li1— O1 | 1.914 (5) | S1—O1—Li1 | 138.73 (17) |
| Li2— O4 | 1.965 (5) | S2—O4—Li2 | 156.2 (2) |

Table S7: Table of crystallographic experimental details.

|  | CuDMAP_2_OTf_2_THF_2_ | DMAPCuN"_2_ | DMAP.LiOTf |
| --- | --- | --- | --- |
| CCDC Number | 2409514 | 2409515 | 9409516 |
| Crystal Data | | | |
| Chemical formula | 0.333(C_24_H_36_CuF_6_N_4_O_8_S_2_) ·0.667(C_23_H_36_CuF_3_N_4_O_5_S) ·0.667(CF_3_O_3_S) | C_19_H_46_CuN_4_Si_4_ | 2(C_8_H_10_F_3_LiN_2_O_3_S)·0.5 (C_6_H_6_) |
| *M*_r_ | 750.23 | 506.50 | 595.41 |
| Crystal system, space group | Monoclinic, *Pc* | Tetragonal, *I*4_1_*cd* | Triclinic, *P*¯1 |
| *a*, *b*, *c* (Å) | 22.9910 (7), 10.3578 (3), 20.7316 (5) | 15.6240 (2), 15.6240 (2), 23.4388 (6) | 5.5636 (2), 13.7181 (4), 18.2094 (7) |
| a, b, g (°) | 90, 101.854 (3), 90 | 90, 90, 90 | 76.973 (3), 84.489 (3), 82.819 (3) |
| *V* (Å^3^) | 4831.7 (2) | 5721.6 (2) | 1340.08 (8) |
| Z | 6 | 8 | 2 |
| m (mm^-1^) | 0.89 | 0.94 | 0.28 |
| Crystal size (mm) | 0.19 × 0.12 × 0.07 | 0.14 × 0.09 × 0.06 | 0.24 × 0.05 × 0.02 |
| Data Collection | | | |
| Absorption correction | Multi-scan | Gaussian | Gaussian |
| *T*_min_, *T*_max_ | 0.702, 1.000 | 0.813, 1.000 | 0.665, 1.000 |
| No. of measured, independent and observed [*I* > 2s(*I*)] reflections | 40811, 16968, 11231 | 31747, 4341, 3906 | 19034, 5459, 3500 |
| *R*_int_ | 0.050 | 0.045 | 0.049 |
| (sin q/l)_max_ (Å^-1^) | 0.625 | 0.714 | 0.625 |
| Refinement | | | |
| *R*[*F*^2^ > 2s(*F*^2^)], *wR*(*F*^2^), *S* | 0.070, 0.198, 1.00 | 0.027, 0.067, 1.05 | 0.046, 0.110, 1.01 |
| No. of reflections | 16968 | 4341 | 5459 |
| No. of parameters | 1292 | 137 | 356 |
| No. of restraints | 17 | 1 | 0 |
| H-atom treatment | Riding | Riding | Riding |
| Dñ_max_, Dñ_min_ (e Å^-3^) | 1.11, -0.50 | 0.77, -0.43 | 0.25, -0.31 |
| Absolute structure | Refined as an inversion twin. | | – |
| Absolute structure parameter | 0.30 (2) | 0.039 (16) | – |

# Autoschlenk

## PCB Control Board


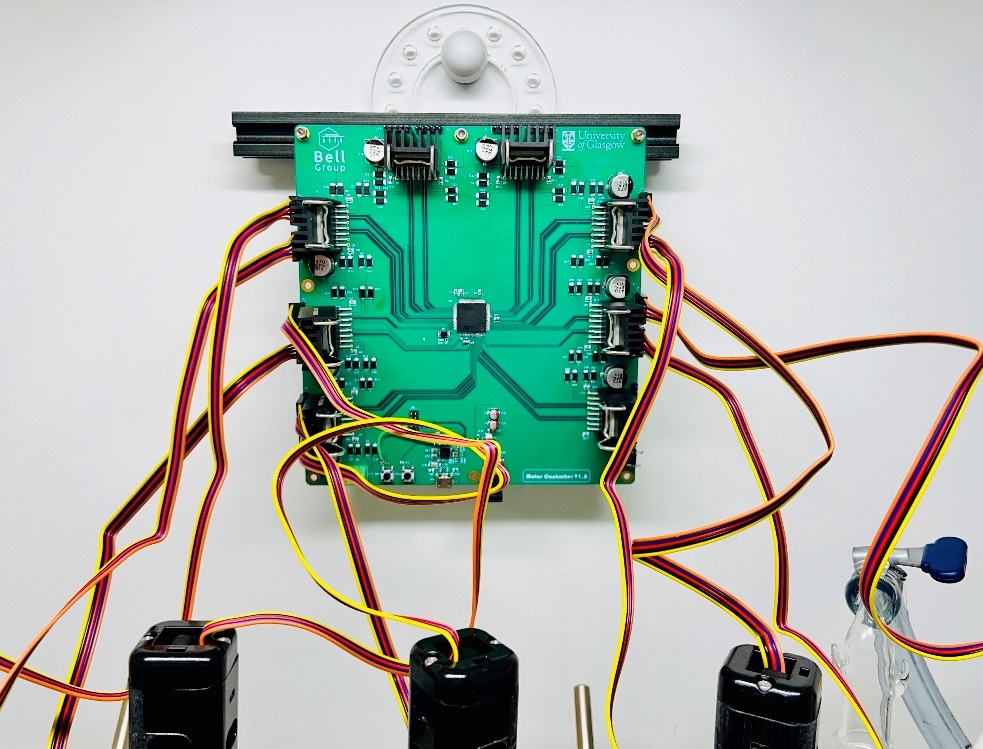
The Autoschlenk is controlled *via* a custom PCB board which is primarily a combination of an ATMEGA 2560 chip with 15 H-bridge motor control drivers. Full details including board schematics and a bill of materials can be found on [GitHub](https://github.com/Bell-Group-Glasgow/autoschlenk) (github.com/Bell-Group-Glasgow/autoschlenk).

Figure S19: Autoschlenk PCB Board.

Original prototyping was conducted using a commercially available Arduino Mega Board appended with L298N Motor Driver boards (each connected to two actuators) connected *via* a breadboard. Since each L298N can drive 2 motors, it is feasible to run the system with one Arduino Mega and five L298N boards; however, to reduce wiring, we have designed our custom board as a combination. Finally, prototype setup requires a voltage divider ([25V Potential Divider Module Resistor Sensor Voltmeter Arduino F – Flux Workshop](https://fluxworkshop.com/products/bdaa100209-25v-potential-divider-blue?srsltid=AfmBOoqMujkeqOGzFxdwdnsALEyaF_hpgT1q3LQO5cFH-fFSfxHmVSY9&variant=32458583441468)) for connection of the vacuum gauge on the line (Leybold Display One).

## Bill of Materials

Table S8: Bill of Materials for the Autoschlenk Line. Note: Does not include vacuum pump, bubbler, gauge, *etc.*

| Part | Supplier | Part Code | Manufacturer Code | Number | Price |
| --- | --- | --- | --- | --- | --- |
| Linear Actuators | RobotShop | RB-Fir-142 | L12-10-210-12-P | 10 | £70.60 |
| o-Rings | RS Components | See Below | | | |
| Board | PCB Online | Custom |  | 1 | £37.47 |
| Board Components | Various (see Bill of Materials) | | |  | £72.40 |
| Raspberry Pi | Farnell | 2842228 | RPI3-MODBP | 1 | £33.07 |
| Touchscreen | RS Components | 899-7466 |  | 1 | £64.15 |
|  | | | | | £913.08 |

## Build Guide for Actuators

Autoschlenk taps consist of linear actuators with internal potentiometer feedback to allow controlled opening and closing of the system to gas and vacuum. In order to attach these to the glass manifold, 3D printed housings, printed using a Stratasys Connex 350 in VeroBlackPlus RGD875 material, were appended with Kalrez o-rings. CAD Files for the 3D printed parts can be found on [Github](https://github.com/Bell-Group-Glasgow/autoschlenk) under ‘Documentation’.

Table S9: Bill of Materials for the Tap Assembly.

| Part | Supplier | Part Code | Number per tap | Price |
| --- | --- | --- | --- | --- |
| Linear Actuators | RobotShop | RB-Fir-142 | 1 | £70.60 |
| o-Rings | RS Components | RS 196 5692/5682 | 3 | £9.41 / 25 |
| Barrell | Custom (3D Printed) | |  |  |
| M3 12mm Screws (Barrell) | RS Components | 281-007 | 1 | £13.82 / 50 |
| M3 Nuts (Barrell) | RS Components | 560-293 | 1 | £5.07 / 250 |
| Actuator Fixing | Custom (3D Printed) | |  |  |
| M2.5 20 mm Screws (Actuator Fixing) | RS Components | 914-1488 | 2 | £5.85 / 100 |
| M2.5 Nuts (Actuator Fixing) | RS Components | 122-4399 | 2 | £3.11 / 100 |
| Actuator Housing | Custom (3D Printed) | |  |  |
| MS Screws (Line Connector) | RS Components | 293-331 | 4 | £20.22 / 50 |
| M3 Nuts (Line Connector) | RS Components | 122-4400 | 4 | £2.90 / 100 |
| Line Connector | Custom (3D Printed) | |  |  |

1. You will need the four 3D printed parts, the actuator, three o-rings and the screws and nuts outlined in the Bill of Materials (Table S8, Figure S20)


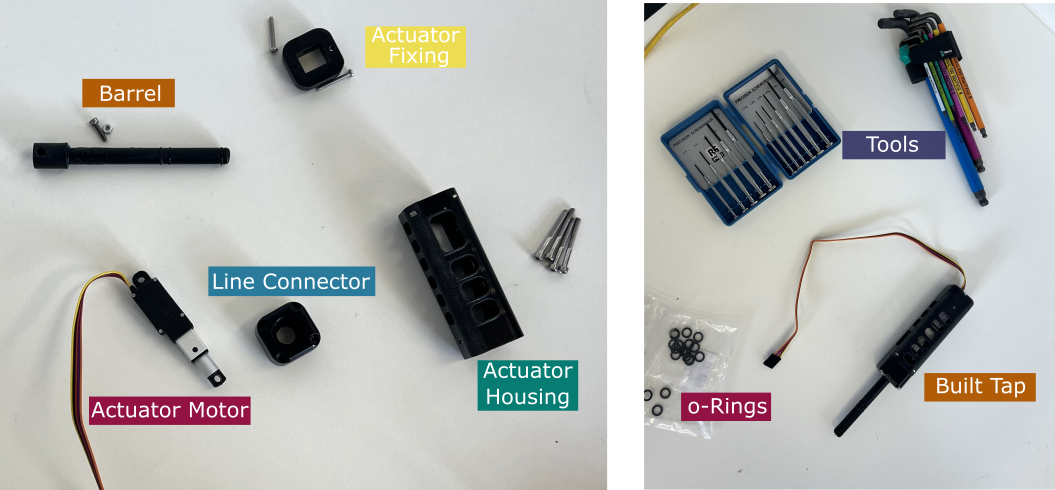


Figure S20: Parts for building Autoschlenk Tap.

1. First affix the o-rings to the three grooves on the barrell. If different sizes of o-rings are required the thicker o-ring must be on the terminal groove. This can vary depending on the glass of the manifold.


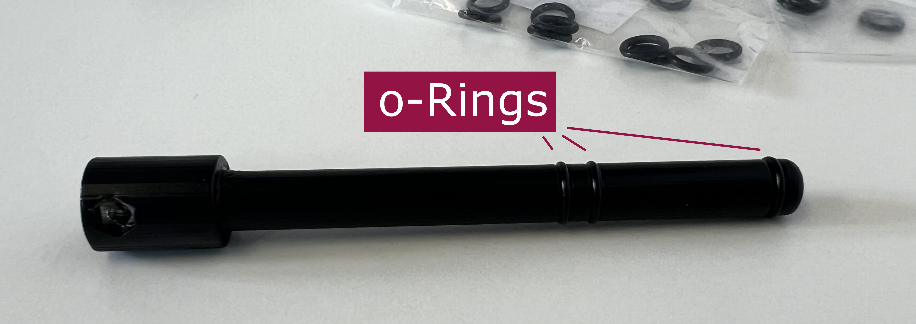


Figure S21: o-rings on the 3D printed barrell.

1. Place the M3 Full profile nut inside the hexagonal cutout on the thicker end of the barrell then thread the barrell through the Line Connector as shown in Figure S22.


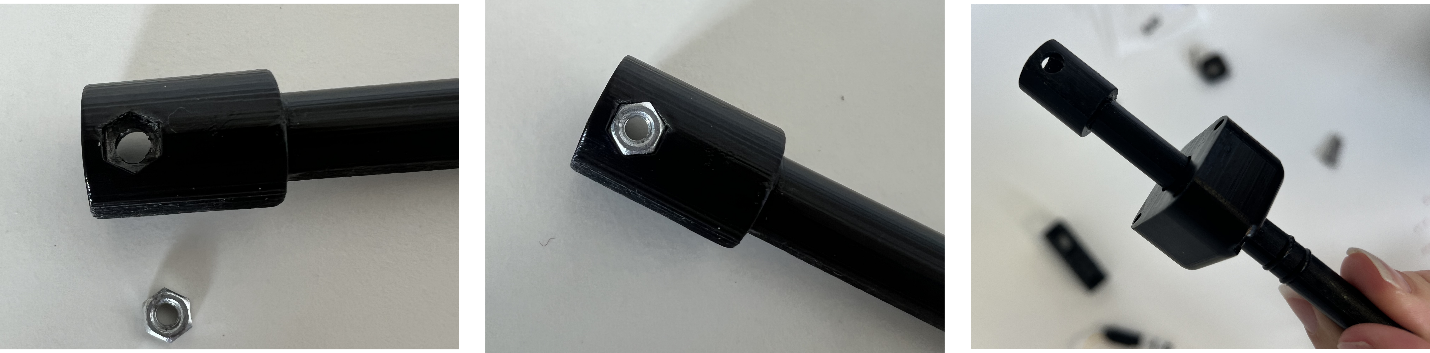


Figure S22: Preparing the barrell for the actuator.

1. Place four M2.5 nuts in the corner cavities of the Actuator Housing print. Connect the Line connector (with barrell) with four M2.5 20mm screws). The barrell move freely for now.


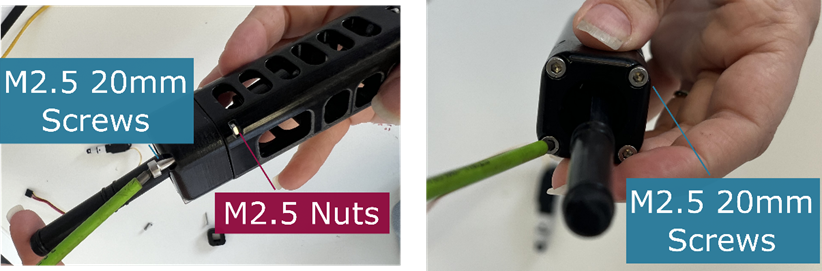


Figure S23: Joining the Line Connector and Actuator Housing Parts

1. Place the Actuator inside the Actuator housing (Figure S24). Note that there is only one possible orientation through which the actuator will fit correctly in the housing. Orient the barrell screw hole towards one of the ‘windows’ in the actuator housing and secure this to the actuator using an M3 12mm screw.


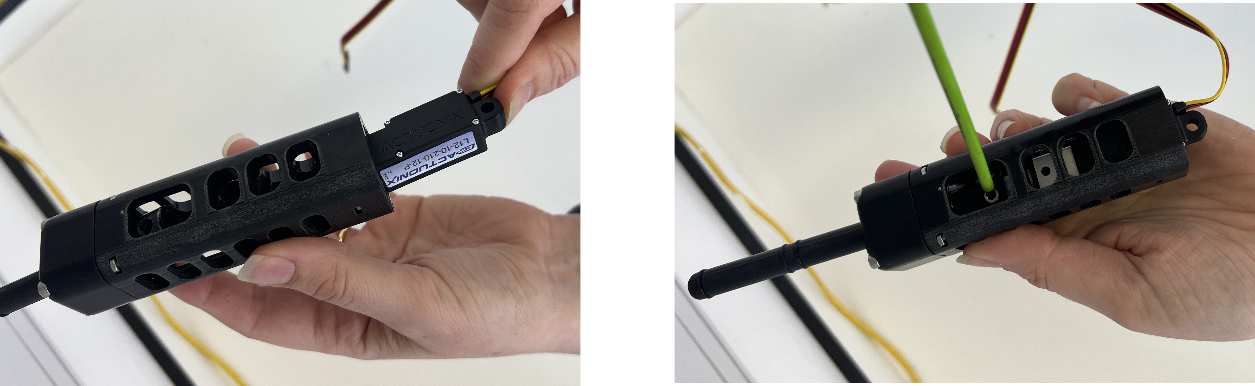


Figure S24: Securing the actuator to the printed barrell.

1. Finally, push two M2.5 nuts through the cavities on opposing faces of the actuator housing. Next, thread the acutator fixing through the wires and secure to the actuator housing with M2.5 20mm screws. Your finished item should look like the Built Tap in Figure S20.


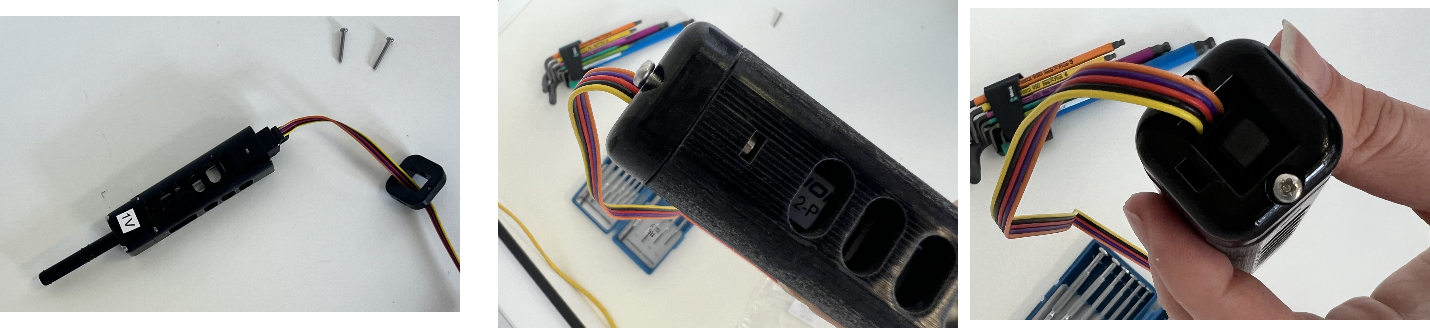


Figure S25: Securing actuator within housing.

## Glass Manifold Build


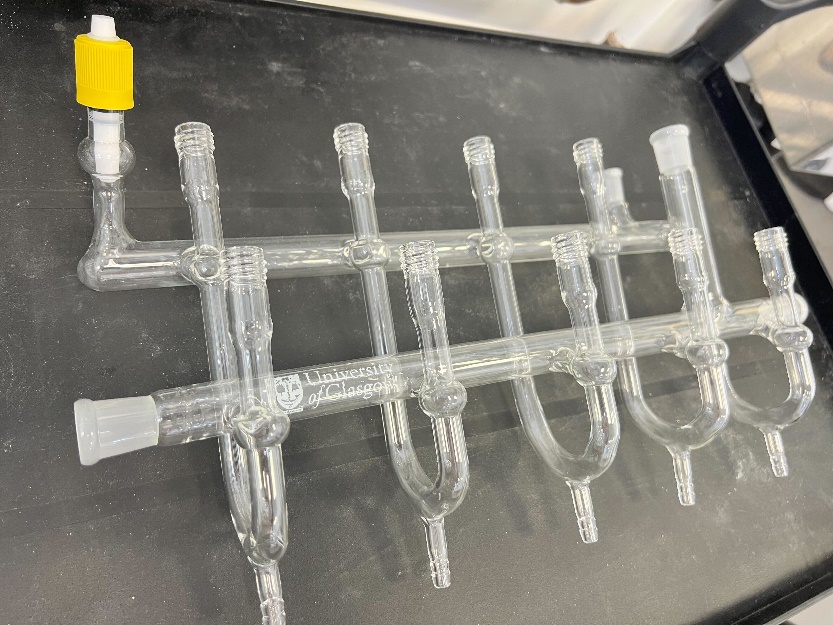
The manifold consists of two main tubes (i.e. gas & vacuum) of 22mm i.d. glass tubing connected by 5 U-bends (with hose barb) which each feature two valves on either side of the U to allow for sealing each line to the primary gas/vacuum tubes.


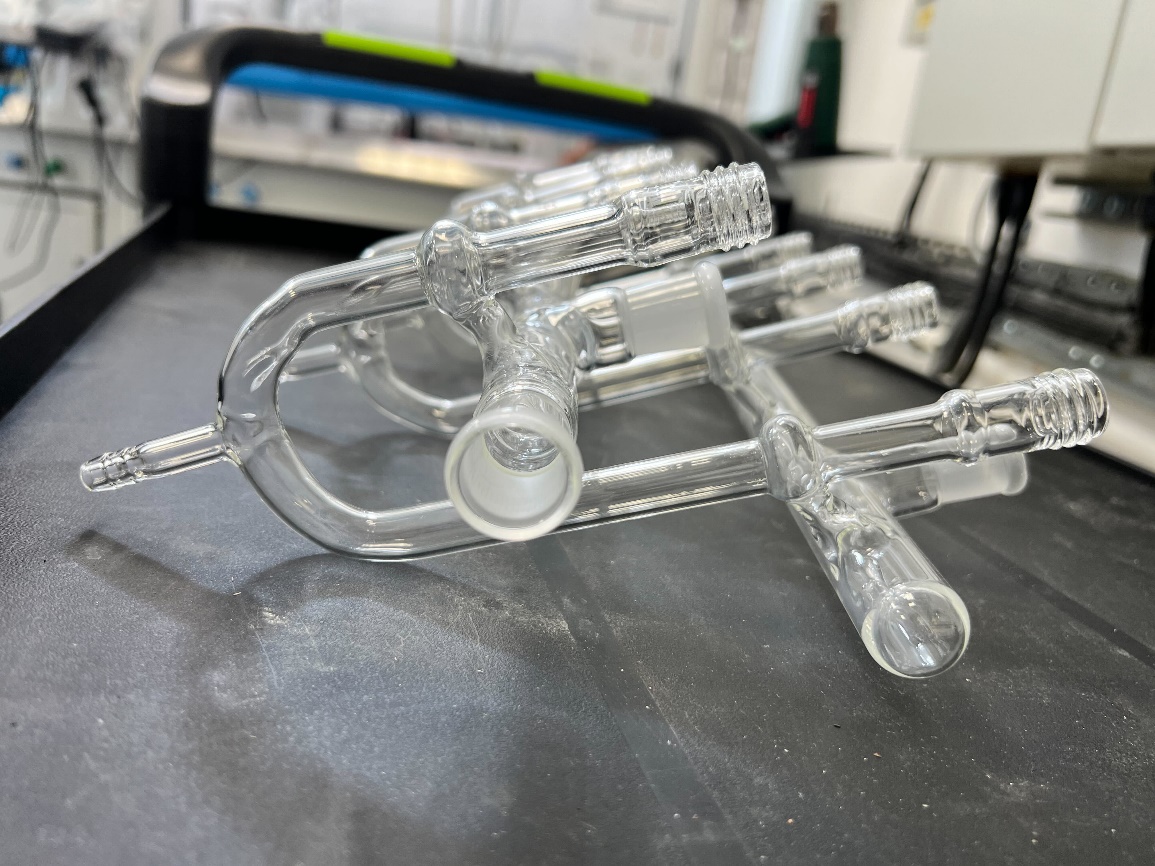
Each valve on the manifold is built by combining a GL18 thread with 10 mm precision bore tubing. The GL18 part extends 30 mm from the top below which the precision tubing is appended. A bulge in the tubing is made below and the bottom line of the bulge in each valve is 75 mm from the top of the GL18. The remainder of the line comprising U-bends to connect the positive/negative pressure manifold to the flexible tubing etc is made from 13 mm medium wall tubing (non-precision) with an internal diameter ca 10mm.


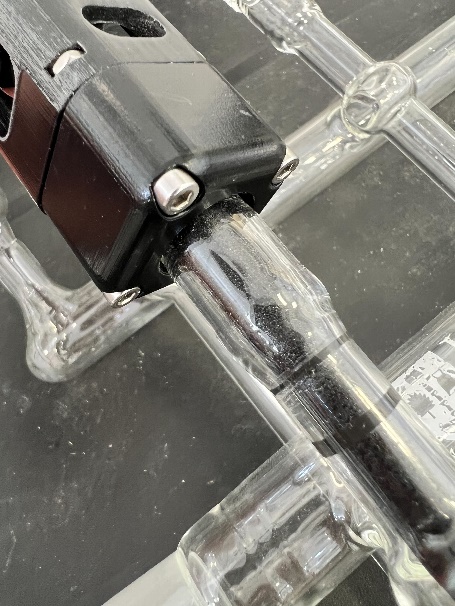

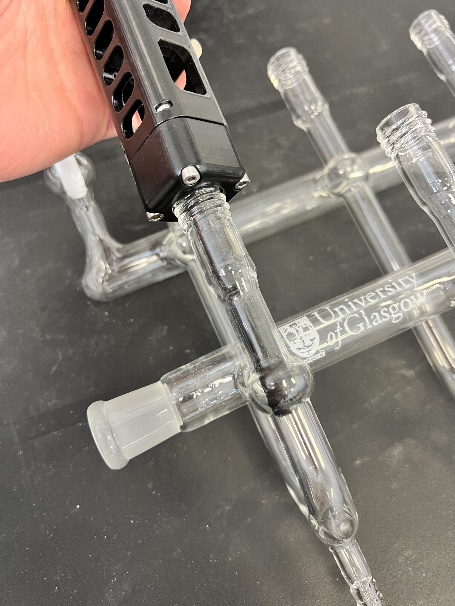

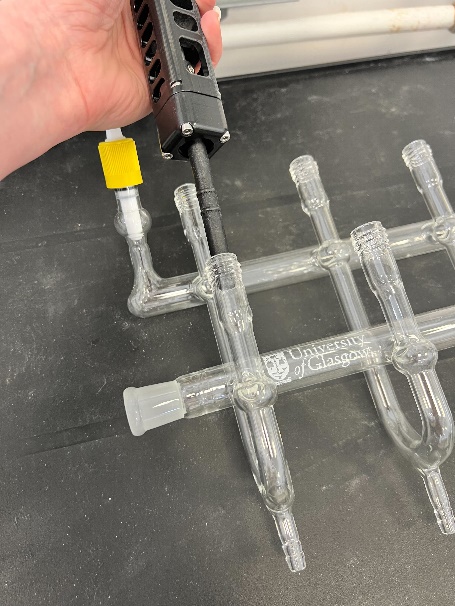


**Figure S26:** Securing tap on Autoschlenk manifold via the 3D printed female GL18 screw thread.

## Code

The firmware for the custom PCB board is written in C++. The Graphical User Interface (GUI) for both PC and Raspberry Pi controlled touchscreen are scripted in Python 3.10. Commands are sent from the Python GUI application to the PCB firmware using serial communication. The stack structure is shown in Figure 1.

Full code can be found at Github.com *via* the following link ([github.com/Bell-Group-Glasgow/autoschlenk](https://github.com/Bell-Group-Glasgow/autoschlenk)). This Autoschlenk repository hosts the code for the PCB firmware, written in C++, and the PC GUI software, written in Python. Alongside this the repository contains a markdown document detailing the installation process alongside the list of dependencies required as well as schematics and a bill of materials for the custom Arduino board.


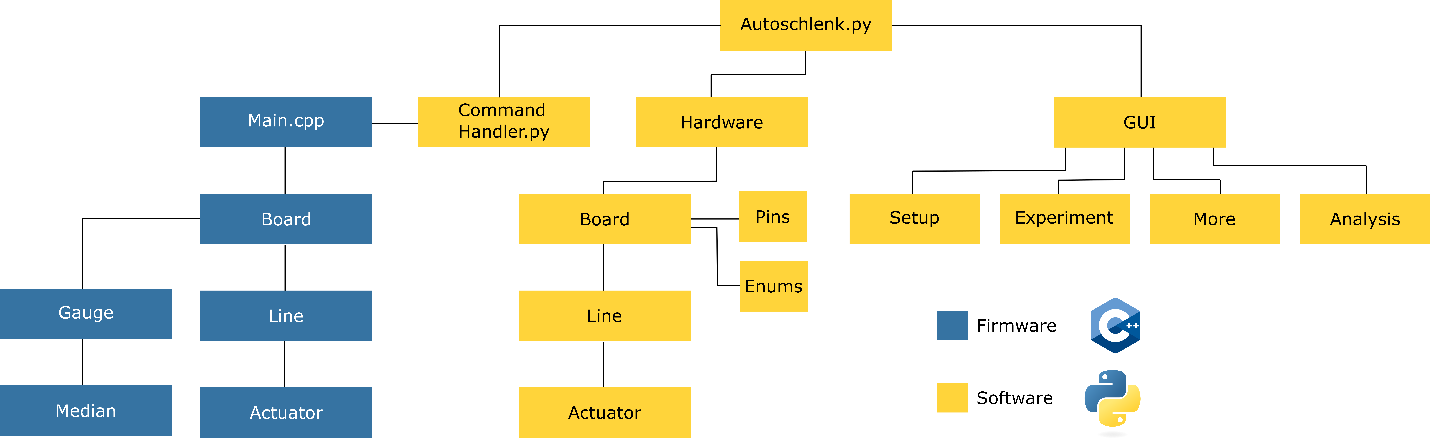


**Figure S27**: Diagram of Stack for Autoschlenk

##

## Depiction of Concentrate Function

Evaporation (Flasks 1-3 Fig.S16):

Evaporation of a solvent from a solution leaves a residue as the meniscus of the solution recedes.

Internal Reflux (Flasks 4-5 Fig.S16):


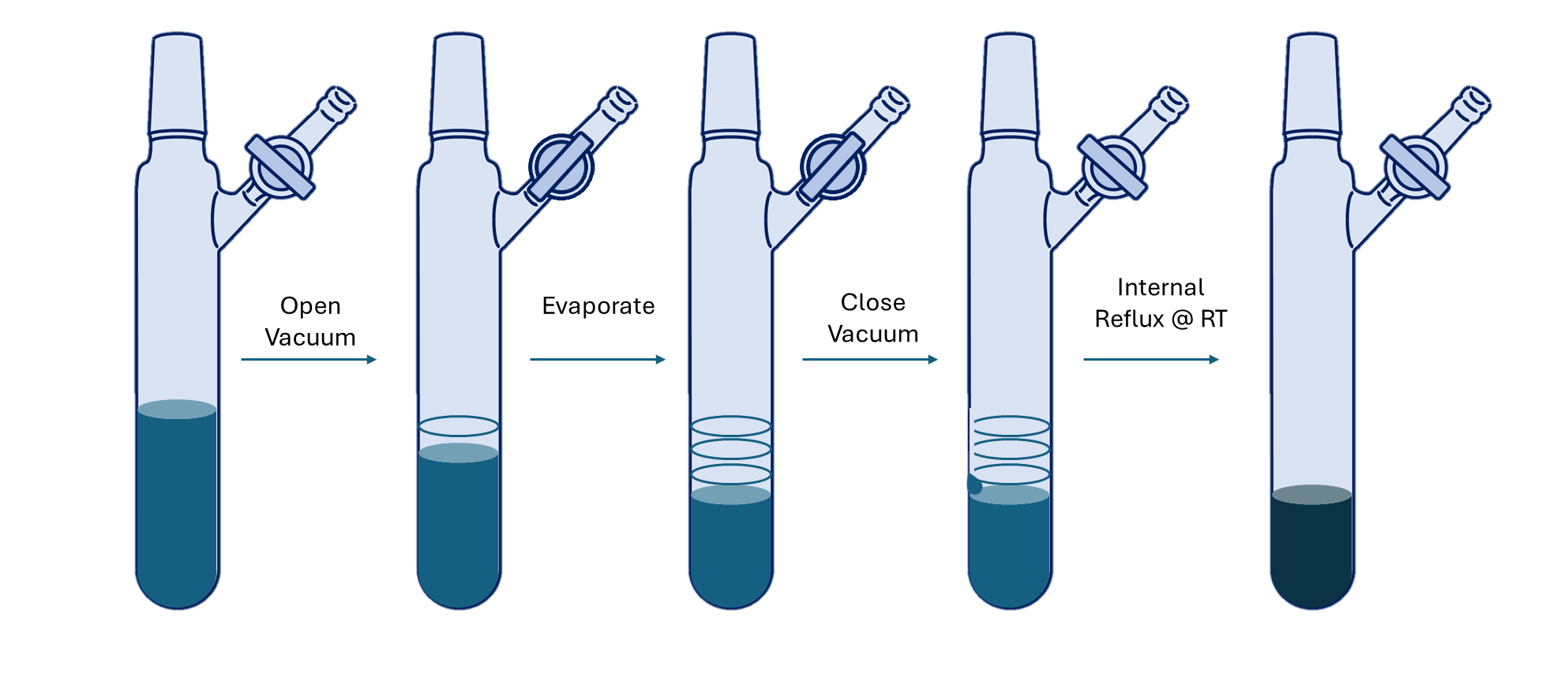
After evaporation, sealing a flask under static vacuum/reduced pressure results in vaporization of some of the solvent (particularly for solvents with low boiling points). This solvent may then condense on the flask above the residue rings and have the effect of washing the solid material back into solution.

**Figure S28:** Visualisation of the concentrate functions effect on a solution.

The Concentrate protocol:


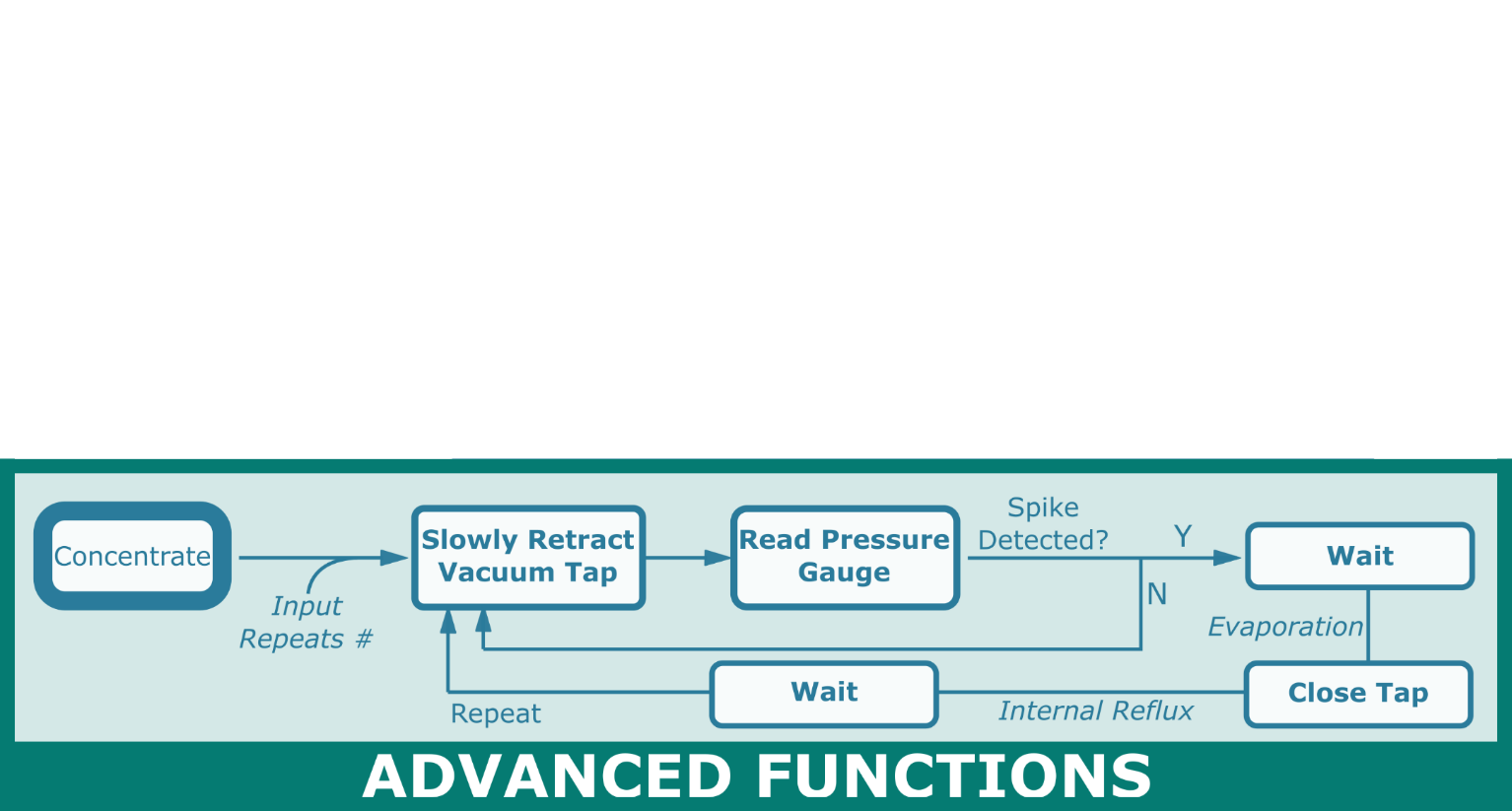
Concentrate first allows for evaporation for a set period by opening the tap on the Autoschlenk line to vacuum. This is then followed by sealing the flask (Close Tap) to allow for internal reflux to take place. Concentration performs these two operations in a repeat loop to allow for maximum supersaturation of the sample. The specific repeat number and time intervals for tap opening and closing are set by the user and may vary depending upon the solvent, volume etc.

**Figure S29:** Concentrate Protocol logic.

**References**

[1] S. Stoll, A. Schweiger,"EasySpin, a comprehensive software package for spectral simulation and analysis in EPR" *Journal of Magnetic Resonance* **2006**, *178*, 42-55.

[2] N. L. Bell, C. Xu, J. W. B. Fyfe, J. C. Vantourout, J. Brals, S. Chabbra, B. E. Bode, D. B. Cordes, A. M. Z. Slawin, T. M. McGuire, A. J. B. Watson,"Cu(OTf)2-Mediated Cross-Coupling of Nitriles and N-Heterocycles with Arylboronic Acids to Generate Nitrilium and Pyridinium Products" *Angew. Chem. Int. Ed.* **2021**, *60*, 7935-7940.

[3] M. J. Frisch, G. W. Trucks, H. B. Schlegel, G. E. Scuseria, M. A. Robb, J. R. Cheeseman, G. Scalmani, V. Barone, G. A. Petersson, H. Nakatsuji, X. Li, M. Caricato, A. V. Marenich, J. Bloino, B. G. Janesko, R. Gomperts, B. Mennucci, H. P. Hratchian, J. V. Ortiz, A. F. Izmaylov, J. L. Sonnenberg, Williams, F. Ding, F. Lipparini, F. Egidi, J. Goings, B. Peng, A. Petrone, T. Henderson, D. Ranasinghe, V. G. Zakrzewski, J. Gao, N. Rega, G. Zheng, W. Liang, M. Hada, M. Ehara, K. Toyota, R. Fukuda, J. Hasegawa, M. Ishida, T. Nakajima, Y. Honda, O. Kitao, H. Nakai, T. Vreven, K. Throssell, J. A. Montgomery Jr., J. E. Peralta, F. Ogliaro, M. J. Bearpark, J. J. Heyd, E. N. Brothers, K. N. Kudin, V. N. Staroverov, T. A. Keith, R. Kobayashi, J. Normand, K. Raghavachari, A. P. Rendell, J. C. Burant, S. S. Iyengar, J. Tomasi, M. Cossi, J. M. Millam, M. Klene, C. Adamo, R. Cammi, J. W. Ochterski, R. L. Martin, K. Morokuma, O. Farkas, J. B. Foresman, D. J. Fox, Wallingford, CT, **2009**.

[4] M. Kaiser, J. Göttlicher, T. Vitova, A. Hinz,"Towards Heteroleptic Dicoordinate CuII Complexes" *Chem. Eur. J.* **2021**, *27*, 7998-8002.
